# *Medicago truncatula* quantitative resistance to a new strain of *Verticillium alfalfae* from Iran revealed by a genome-wide association study

**DOI:** 10.3389/fpls.2023.1125551

**Published:** 2023-04-14

**Authors:** Amir Hossein Fartash, Cécile Ben, Mélanie Mazurier, Asa Ebrahimi, Mojtaba Ghalandar, Laurent Gentzbittel, Martina Rickauer

**Affiliations:** ^1^ Laboratoire écologie fonctionnelle et environnement, Université de Toulouse, Centre National de Recherche Scientifique, Toulouse Institut National Polytechnique, Université Toulouse 3 – Paul Sabatier (UPS), Toulouse, France; ^2^ Project Center for Agro Technologies, Skolkovo Institute of Science and Technology, Moscow, Russia; ^3^ Department of Plant Breeding and Biotechnology, Science and Research Branch, Islamic Azad University, Tehran, Iran; ^4^ Plant Protection Department, Markazi Agricultural and Natural Resources Research and Education Center, Arak, Iran

**Keywords:** alfalfa, biotic stress, fungal pathogen, gene expression, global warming, legume, vascular wilt, quantitative disease resistance

## Abstract

Verticillium wilt is a major threat to many crops, among them alfalfa (*Medicago sativa*). The model plant *Medicago truncatula*, a close relative of alfalfa was used to study the genetic control of resistance towards a new *Verticillium alfalfae* isolate. The accidental introduction of pathogen strains through global trade is a threat to crop production and such new strains might also be better adapted to global warming. Isolates of *V. alfalfae* were obtained from alfalfa fields in Iran and characterized. The Iranian isolate AF1 was used in a genome-wide association study (GWAS) involving 242 accessions from the Mediterranean region. Root inoculations were performed with conidia at 25°C and symptoms were scored regularly. Maximum Symptom Score and Area under Disease Progess Curve were computed as phenotypic traits to be used in GWAS and for comparison to a previous study with French isolate V31.2 at 20°C. This comparison showed high correlation with a shift to higher susceptibility, and similar geographical distribution of resistant and susceptible accessions to AF1 at 25°C, with resistant accessions mainly in the western part. GWAS revealed 30 significant SNPs linked to resistance towards isolate AF1. None of them were common to the previous study with isolate V31.2 at 20°C. To confirm these *loci*, the expression of nine underlying genes was studied. All genes were induced in roots following inoculation, in susceptible and resistant plants. However, in resistant plants induction was higher and lasted longer. Taken together, the use of a new pathogen strain and a shift in temperature revealed a completely different genetic control compared to a previous study that demonstrated the existence of two major QTLs. These results can be useful for *Medicago* breeding programs to obtain varieties better adapted to future conditions.

## 1 Introduction

Plants are continuously in contact with a myriad of microorganisms of which some are pathogenic. However, thanks to their innate immunity system, disease is rather the exception than the rule, at least in undisturbed environments. Disease resistance in plants has been described as qualitative (complete, gene-for-gene) disease resistance (Flor, 1971) and quantitative (partial) disease resistance (QDR) (Poland et al., 2009). Qualitative resistance which is governed by a single gene can be neutralized easily by the evolution of new pathogen strains (Flor, 1971; Poland et al., 2009). QDR which is controlled by the contribution of multiple genes of (usually) small effect and their cumulative actions is characterized by a continuous phenotypic variation among populations, from total resistance to high susceptibility (Poland et al., 2009) and varies with environmental conditions (Bartoli and Roux, 2017). Due to the polygenic heredity QDR is more durable (Roumen, 1994).

Plants’ defense mechanisms and pathogens’ pathogenicity have undergone a series of adaptive changes during co-evolution. When a pathogen overcomes preformed defense structures, the plant’s innate immune system is activated by recognition of conserved pathogen-associated molecular patterns (PAMPs) also named microbe-associated molecular patterns (MAMPs) (Ausubel, 2005) or damage-associated molecular patterns (DAMPs) through pattern recognition receptors (PRRs) (Bigeard et al., 2015; Miller et al., 2017). The detection of MAMPs/PAMPs and DAMPs triggers an array of defense responses known as pathogen-triggered immunity, PAMP-triggered immunity (PTI), or MAMP-triggered immunity (MTI) (Bigeard, Colcombet and Hirt, 2015). PTI/MTI involves the production of antimicrobial compounds and pathogenesis-related (PR) proteins in the plant (Bigeard et al., 2015; Miller et al., 2017; Kamle et al., 2020).

As adaptive response pathogens evolved to produce and release effectors into the host plant cells which suppress PTI/MTI. The plants’ response was to adapt their immune system by recruiting a second layer of defense that directly or indirectly detects pathogen effectors through plant resistance (R) proteins leading to Effector-Triggered Immunity (ETI) (Zipfel, 2014; Miller et al., 2017; Kamle et al., 2020).

Recognition of the pathogen through perception of PAMPs/MAMPs, in the case of PTI/MTI or effectors, in the case of ETI, is followed by cascades of signaling pathways such as ion fluxes and phosphorylation of proteins (Shen et al., 2017) leading finally to defense mechanisms such as strengthening of cell walls (Vogel and Somerville, 2000; Zhang et al., 2022), production of reactive oxygen species (ROS), pathogenesis related proteins (PRs) and phytoalexins (Wojtaszek, 1997; Okada et al., 2015; Rout et al., 2016), either locally or systemically.

This fragile balance between plants resistance and pathogens’ virulence is more and more threatened by anthropogenic factors such as global trade. The exchange of seeds and plants worldwide has led to the spread of pathogens in areas where they were absent before and to which local plants may not have evolved resistance mechanisms.

Verticillium wilt is a vascular disease caused by the soil-borne fungus *Verticillium* spp. This disease is one of the most destructive fungal diseases in the world and affects more than 200 different hosts, among them many economically important crops (Klosterman et al., 2011). It is found mainly in temperate regions, but can also occur in hotter climates (Erwin and Howell, 1998; Klosterman et al., 2011). The fungus enters the roots of its host plants through natural cracks or wounds and colonizes the xylem vessels which leads to their plugging through the production of gels in susceptible hosts (Cooper and Wood, 1980; Fradin and Thomma, 2006; Inderbitzin et al., 2014). Visible symptoms are yellowing, wilting and finally death of the plant. *Verticillium* species are able to survive in the soil for many years (Pegg and Brady, 2002; Agrios, 2005) through the production of thick-walled pigmented resting structures. This feature greatly reduces the possibilities of disease management (Inderbitzin et al., 2011). The best strategy so far is breeding of resistant crop varieties.

*Verticillium dahliae* is the most important and best studied *Verticillium* species due to the high number of its host plants and the economic impact of the disease (Klosterman et al., 2011; Inderbitzin and Subbarao, 2014). *V. alfalfae* which has a narrower host range and is most aggressive on alfalfa is a major threat to this important forage crop worldwide (Acharya and Huang, 2003; Graham and Vance, 2003). The tetraploid and outcrossing nature of alfalfa makes genetic studies of disease resistance difficult, but synteny and sequence homologies with model plants such as *Medicago truncatula* can be of great help.

*Medicago truncatula*, a diploid autogamous wild plant and close relative of alfalfa, has been established as a model plant for legume crops (Cook, 1999). It is native to the Mediterranean region, presents high biodiversity, and many genomic and genetic resources are available (Ellwood et al., 2006; Gentzbittel et al., 2015). It is a host to *V. alfalfae* and quantitative resistance to this fungus relying on several QTLs has been reported (Ben et al., 2013). Resistant plants were shown to stop fungal colonization of their roots at early stages, and exhibited transcriptional responses related to innate immunity (Toueni et al., 2016). Based on the interaction between *M. truncatula* and *V. alfalfae* our group has studied the link between genome admixture and resistance to this pathogen, using geographical origin of plant accessions as covariates (Gentzbittel et al., 2019).

This study revealed that resistant accessions were mostly found in populations from the western part of the Mediterranean basin, with a gradient of susceptibility to resistance from east to west. This led to the hypothesis that host plant and pathogen strain may have co-evolved, and that an isolate from the east might reveal a different pattern of the plant’s genetic control of resistance.

To test this hypothesis a new *Verticillium alfalfae* isolate was obtained from Iran, the far eastern part of the plant’s natural habitat. Inoculations were performed at 25°C, in agreement with the pathogen’s optimum temperature for sporulation and growth, and the geographical distribution of resistance and susceptibility was plotted.

*Loci* involved in the plant’s response were identified by a genome-wide association study (GWAS) taking advantage of the international MtHapMap project (Tang et al., 2014) which provides genomics data on a large number of accessions.

Results were compared to those obtained in a previous GWAS study, with a French *V. alfalfae* isolate and at 20°C. A high correlation between the two studies was observed concerning the geographical distribution of resistant and susceptible accessions. However no common *loci* associated to resistance were detected by SNPs. The expression study of some genes underlying the *loci* showed that they were all expressed in roots and induced by inoculation with *V. alfalfae*.

## 2 Material and methods

### 2.1 Fungal isolates

#### 2.1.1 Isolation of fungal strains and selection of *Verticillium alfalfae*

**Figure 1:**
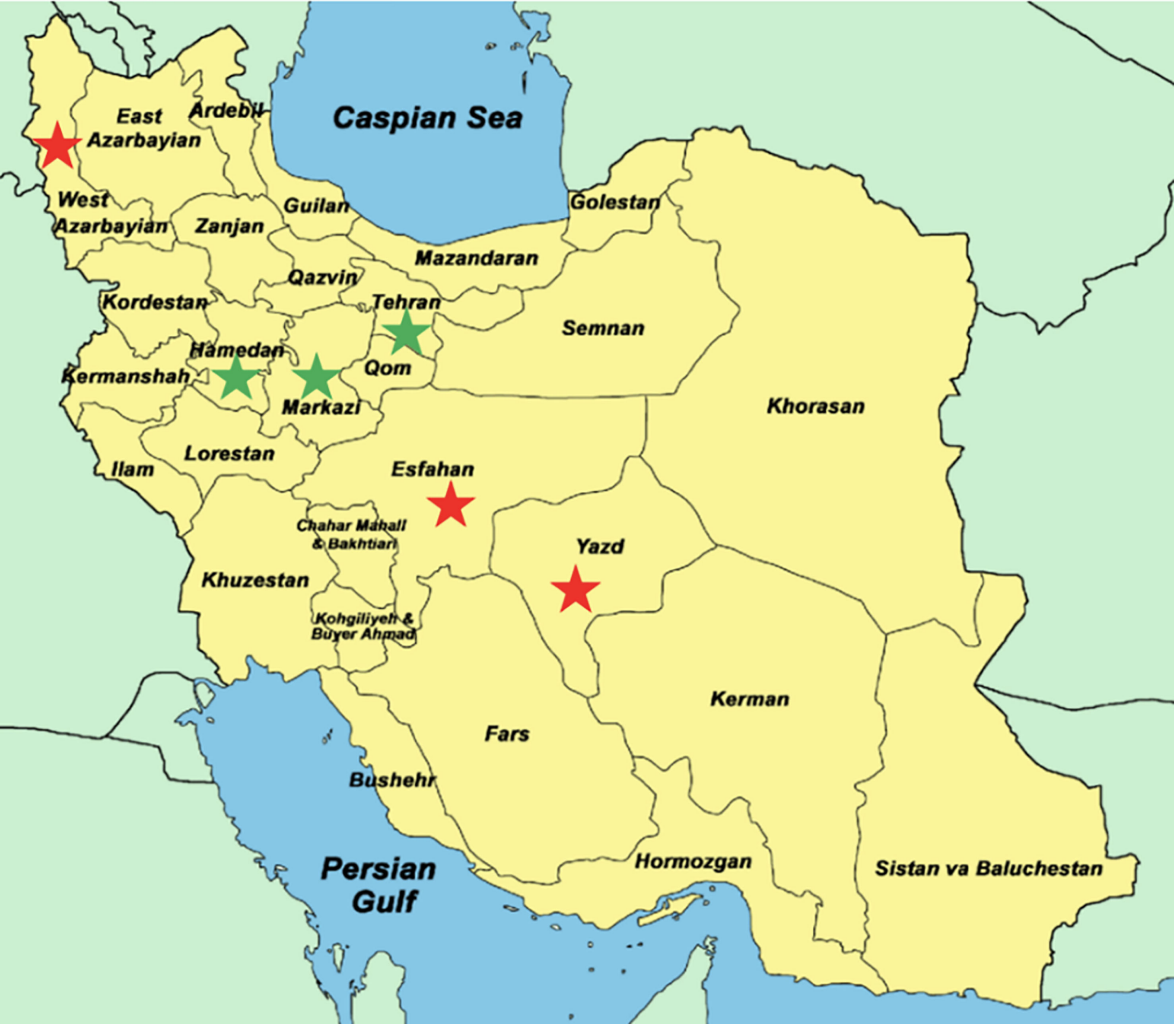
Map of Iran showing provinces where alfalfa plants with wilting symptoms were collected for fungus isolation. The green stars show the provinces where fungal isolates were confirmed by PCR to be *V. alfalfae*, the red stars show provinces where the fungal isolates were not confirmed as *V. alfalfae*. Map adapted from Pešić et al. (2014).

Symptomatic alfalfa plants were collected from six different regions in Iran (**Figure 1**) and dried between paper for conservation. Their stems were cut into 2 cm long fragments above the first node and after surface sterilization (15 sec in 70% ethanol and 6 minutes in 0.96% commercial bleach), the fragments were incubated on PDA containing 50 µg/ml streptomycin and incubated at 25°C (Mazurier, 2018). After 3 days, outgrowing mycelium was subcultured on fresh PDA, and purified by further subculturing. Monospore cultures were prepared when isolates were pure by visual assessment. The isolates were cultured on water agar at 25°C for observation of conidiophores under the microscope. Samples exhibiting the characteristic verticillate form of conidiophores were considered as *Verticillium* sp. and were retained for further identification steps.

#### 2.1.2 Molecular identification of fungal isolates

The fungal isolates were grown in Potato Dextrose Broth (PDB) for 2 weeks and the mycelium was harvested by filtration and stored at −20°C.

DNA was extracted from frozen mycelium using the CTAB protocol (Carter-House et al., 2020). The quantity and quality of DNA was assessed with a nanodrop (NanoDrop nd-1000 Spectrophotometer).

Molecular identification was performed by PCR using the species-specific primers AlfF/AlfD1r (for *V. alfalfae*) and NoF/NoNuR (for *V. nonalfalfae*) (Inderbitzin et al., 2013). The ITS universal fungal primers (White et al., 1990) were used as a quality control (**Supplementary Table 1**). The PCR reaction mix contained: 1X PCR buffer, 2 mM MgCl2, 25 µM dNTPs, 1 µM each primer, 1.4 U Taq polymerase. Amplification was performed in 30 reaction cycles (1 min at 94°C, 1 min at 50°C for ITS primers, 62°C for AlfF/AlfD1r and 65°C for NoF/NoNuR primers, and 2 min at 72°C) after denaturation for 10 min at 94°C, and was followed by extension for 10 min at 72°C. Amplicons were electrophoresed on a 1% and 1.5% agarose gel containing Ethidium bromide, for amplification with ITS1-ITS4 and species-specific primers respectively. DNA bands were visualized through the Quantum st5 gel documentation system.

#### 2.1.3 Analysis of *Verticillium alfalfae* growth and sporulation

Small disks (0.8 cm) of mycelium were punched from the border of 2-week-old cultures and inoculated in the center of Petri dishes containing 15 ml PDA. The diameter of the colony was measured at regular intervals for 2 weeks at 20°C, 25°C, and 28°C in the dark.

After 14 days, 20 mL of sterile water was added to every culture and the surface of the mycelium was rubbed gently with a bent Pasteur pipette to release the conidia. The conidia were collected and their concentration was determined under the microscope with a Malassez counting chamber.

The study was performed in three independent experiments including two independent blocks through augmented split-plot design where the whole-plot factor was assigned to temperature (three separate incubators) and split-plot factor was assigned to the fungal strains. In each experiment three Petri dishes were used per strain per condition.

The linear mixed model was used to analyze the effect of temperature on hyphal growth and sporulation as follows:


(Equ. 1) Y=Xb+Zu+e

where Y is the response vector (observed values of hyphal growth or spore production) X is the N x p design matrix for the p fixed factor including the grand mean of the trait (hyphal growth or spore production), temperatures and the different strains, and Z represents N x q_j_ design matrix for the q random effects of experiments (defined as the combination of blocks within repeats, repeats and temperature).

An analysis of variance (ANOVA) was performed using the lmer function of the lmerTest package (Kuznetsova et al., 2017) of the R 4.1.0 statistical software (R Core Team, 2020) to determine variability for growth and sporulation among the *V. alfalfae* isolates. The least square means (LSmeans) (Lenth, 2013) for each strain were computed and the mean comparison grouping was performed by the Tukey method.

### 2.2 Plant material and genome-wide association mapping

#### 2.2.1 Plant growth, inoculation and phenotyping

The MtHapMap collection was multiplied in our greenhouse in 2014 for a GWAS study at 20°C (Mazurier, 2018). A set of 242 *Medicago truncatula* accessions was selected based on the number of available seeds (**Supplementary Table 2**) for the present study.

They were scarified manually with sandpaper and incubated at 5°C in Petri dishes between layers of moist filter paper for 2–3 days, then were transferred to room temperature for 24h. Germinated seeds were transplanted into Jiffy substrate (jiffy^®^-7 diameter 33cm) and seedlings were grown in a phytotron with 25°C day/23°C night and a photoperiod of 16h.

Ten-day-old seedlings were subjected to root inoculation with conidia of isolate AF1 at a concentration of 10^6^ spores/ml and symptom development was scored regularly on a scale from 0 to 4, as described by Ben et al. (2013) during 4 weeks under the same conditions as for growing.

Disease intensity and progress were evaluated through Maximum Symptom Scores (MSS, the score on the last day of symptom scoring) and Area Under the Disease Progress Curves (AUDPC) respectively.

In total, 8,442 inoculated plants were assessed through an augmented block design with three independent experiments for all 242 accessions; 4 accessions (F83005.5, DZA315.16, DZA45.5, and A17) were included in each block as check lines to evaluate the block effects. Each experiment contained 6–12 plants per accession, while the check accessions were consistently six plants per block.

#### 2.2.2 Data analysis

For data analysis the Mixed Linear Model (MLM) approach was applied. Estimated breeding values corresponding to the traits (AUDPC and MSS) were calculated as BLUEs (Best Linear Unbiased Estimation) through the following mixed linear model:


(Equ. 2) Y=Xb+Zu+e

The BLUEs were extracted from the model to be used for genome-wide association mapping.

Data transformation was deployed whenever it was needed to homogenize variances and normalize residuals of the ANOVAs.

Multiple mean comparisons were performed using Tukey (pairwise comparisons) tests at p-value ≤ 0.05 using the cld function of the multcomp R package to group the accessions with regard to their response to inoculation with the selected Iranian *V. alfalfae* isolate.

The broad sense heritability (H^2^) was calculated through the variance components method on raw phenotypic data while the blocks and repeats were regarded as fixed effects and the accession regarded as random effect.

For comparison of the results of the current study with a previous one performed with the French *V. alfalfae* strain V31.2 at 20°C, the extracted BLUE values from the adjusted mean values of MSS were used, and the correlation among them was calculated.

#### 2.2.3 Genome-wide association mapping

To fine map the genomic regions of *M. truncatula* with additive effects associated with AUDPC and MSS, TASSEL 5.2.50 was used (Bradbury et al., 2007). Single Nucleotide Polymorphism (SNP) data were obtained from the *Medicago truncatula* HapMap project (http://www.medicagohapmap.org/) and were filtered with a minimum allele frequency (MAF) of 5% and minimum count to 200, which resulted in 5,671,743 SNPs retained.

A total of five GWAS statistical models were tested including General Linear Model (GLM) without any correction for population structure (naive model), GLM Q-Model with Q-matrix as correction for population structure and three Mixed Linear Models (K model, Q model and K + Q model) with K-matrix and/or Q-matrix respectively as correction for kinship relationships and population structure. The population structure (Q matrix) is based on population admixture proportions of *M. truncatula* individuals. The kinship matrix (K matrix) was computed from a 840K LD-pruned SNP dataset. Both matrices have been described earlier by Gentzbittel et al. (2019). To reduce computing time, the Population Parameter Previously Determined (P3D) algorithm (Zhang et al., 2010) was used to fit mixed linear models.

For all models, the Q-Q plot was plotted to evaluate which was the best fitted model for this study. Manhattan plots were computed to illustrate the association of SNPs with AUDPC and MSS.

For plotting the Manhattan graph the association score of SNPs was calculated through p-values of all SNPs as follows - log10(p-values).

To correct for multiple testing, Bonferroni and False Discovery Rate (FDR) corrections were used. Multiple testing adjustment was conducted at α = 0.05.

### 2.3 Gene expression studies

#### 2.3.1 Selection of candidate genes and primer design

The suggestive line which was proposed by the qqman R package (D. Turner, 2018) was very close to the FDR, thus it was chosen as the main threshold for selecting the significant candidate SNPs. Hence, SNPs with association score values equal or greater than 5 were selected. Based on data on linkage disequilibrium and recombination rates described for *M. truncatula* in a previous study (Branca et al., 2011), the regions 10 kb upstream and downstream of these SNPs were explored on the JBrowser site (Buels et al., 2016). Genes with functional annotation associated to defense pathways which were located within these areas were favoured as possible candidate genes. In addition, two genes encoding hypothetical proteins were included for further steps because of their high association score.

The full sequence of selected candidate genes was obtained from the MtHapMap site (http://www.medicagohapmap.org/fgb2/gbrowse/mt40/). Primers for quantitative real-time PCR (qRT-PCR) were designed using the primer3plus web interface for primer3 (Primer3Plus Version: 2.4.2, https://primer3plus.com/cgi-bin/dev/primer3plus.cgi). For each candidate gene, primer pairs were designed based on exon-exon junction and were examined for primer stability (https://www.ncbi.nlm.nih.gov/tools/primer-blast/) and specific amplification through blast on the *M. truncatula* A17 genome (http://www.medicagohapmap.org/tools/blastform; https://blast.ncbi.nlm.nih.gov/Blast.cgi?PROGRAM=blastn&PAGE_TYPE=BlastSearch&LINK_LOC=blasthome). The primer pairs for qRT-PCR were selected based on the results of primer efficiency tests with a mix of cDNA from all conditions and time points (**Supplementary Table 3**).

#### 2.3.2 Inoculation of plants and quantitative real-time PCR

Gene expression studies were performed in three independent experiments. Twelve of the most susceptible and resistant accessions based on the Tukey’s multiple comparison of BLUEs values of AUDPC were selected (**Supplementary Table 4**). Germinated seedlings were transferred to plug trays containing a mixture of sand-perlite (2/3 sand, 1/3 perlite), and grown for 10 days in a phytotron under the same conditions as described above.

Root inoculation and maintenance of the inoculated seedlings was performed under the same conditions as described for phenotyping before.

Roots and aerial parts of plants were harvested separately at 0, 4, 24 and 96 hours post inoculation (hpi) and pooled into a resistant and a susceptible group for each harvesting time. Total RNA was extracted using 200 mg of frozen root tissue samples following the TRIzol method (Invitrogen). After DNase I treatment (Promega) RNA quality was analyzed by NanoDrop nd-1000 Spectrophotometer and subsequently purified by the LiCl precipitation method (Cathala et al., 1983).

cDNA was synthesized from one microgram of pure RNA with the ImProm-II ™ Reverse Transcription System kit (Promega, A3800) using Oligo (dT)_15_ primer following the manufacturer’s instructions.

qRT-PCR was performed with the EurobioGreen^®^ mix qPCR 2X Lo-Rox Kit (Eurobio Scientific, Reference GAEMMX02L-8T) in the QuantStudio™ 6 Flex Real-Time PCR System (Applied Biosystems). At least two technical replicates were run for all samples.

The reaction consisted of one step of 95°C for 3 min for cDNA denaturation, followed by 40 cycles of denaturation and annealing/polymerization (95°C/15sec, 60°C/30 sec). To assess the quality of qRT-PCR reactions the melting curve was implemented at the end of the reaction (95°C/15 sec, 60°C/15 sec, 95°C/15 sec). In order select the primers for gene expression studies we checked primer efficiency with a dilution series of an equimolar mix of cDNA from mock-inoculated and inoculated roots of the susceptible and resistant plants.

The C_T_ values were extracted through Design & Analysis software (V.2.4.3). Relative gene expression levels were analyzed by the comparative C_T_ method (Schmittgen and Livak, 2008). The Medtr2g008050 (Actin) and Medtr4g097170 (H3L) housekeeping genes were used for normalization. ΔC_T_ values were normalized against the harmonic mean of the two housekeeping genes.

## 3 Results

### 3.1 *Verticillium alfalfae* isolates from Iran differ from the French strain V31.2

In order to obtain *V. alfalfae* strains from a region east of the Mediterranean basin with higher temperatures than France, samples from alfalfa fields exhibiting typical wilting symptoms were collected during two field trips in Iran (**Figure 1**).

Putative *V. alfalfae* isolates were obtained from diseased alfalfa plants and after visual assessment sixteen were further analyzed by PCR with *V. alfalfae*-specific (AlfF/AlfD1r) and *V. nonalfalfae*-specific (NoF/NoNuR) primers. Amplification with AlfF/AlfD1r resulted in a band of the expected size of 1060 bp (Inderbitzin et al., 2013) for ten Iranian isolates and the French strain V31.2. No band was obtained with the negative controls *V. dahliae* strain JR2 and *V. non-alfalfae* strain LPP0323 (data not shown). Inversely, PCR with the *V. non-alfalfae* specific primers amplified only DNA from strain LPP0323 but not from Iranian isolates and the French strain V31.2 (data not shown).

In contrast to the French isolate, discoloration and formation of dark sections (black resting structures) were observed for the Iranian isolates and they occurred more frequently at 25°C than at 20°C.

**Figure 2:**
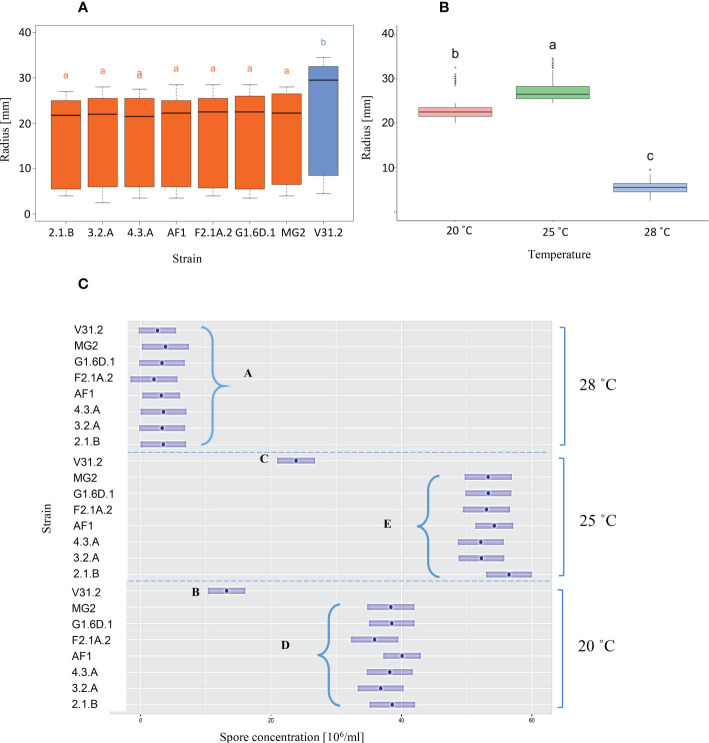
Radial gowth and sporulation of *V. alfalfae* strains at 20 °C, 25 °C and 28 °C. Fungal isolates were cultured on PDA in darkness and hyphal growth was measured as radius during 14 days at regular intervals. Spores were harvested after 14 days. **(A)** Boxplot of strains’ effect on radius after 14 days of growth, **(B)** Boxplot of temperatures’ effect on radius after 14 days of growth. **(C)** Spore production as function of strain and temperature. The letters represent distinct groups by LSmeans based on the Tukey’s test. The experiment was performed in 3 independent repetitions, each with 3 Petri dishes per condition and strain.

To investigate vegetative and reproductive features of the Iranian isolates, hyphal growth and the amount of produced conidia were examined at three different temperatures (**Figure 2**).

Radial growth of seven Iranian *V. alfalfae* isolates and the French strain V31.2 on PDA medium showed similar behavior for all isolates with linear growth for up to two weeks. Growth was best at 25°C and very poor at 28°C (**Supplementary Figure 1**). Compared to the French strain, the Iranian isolates grew less at all three temperatures.

Statistical analysis by ANOVA confirms a significant effect of both strain and temperature on growth rate (**Table 1**). The Iranian strains belong to one group and the French strain belongs to a distinct group for vegetative growth (**Figure 2A**), as revealed by Tukey multiple means comparison test. Twenty-five °C was the best temperature for all isolates, followed by 20°C (**Figure 2B**).

**Table 1:** Analysis of Variance of the effect of temperature and fungal strain on vegetative growth and sporulation.

Type III Analysis of Variance Table with Satterthwaite’s method							
		Radial Growth			Sporulation		
Source of variation	NumDF	Mean Sq	F value	Pr(>F)	Mean Sq	F value	Pr(>F)
**Strain**	7	292.4	8.33	1.25e-08 ***	708	106.1	2e-16 ***
**Temperature**	2	19344.0	551.56	2.2e-16 ***	8531	1278.3	8.3e-16 ***
**Strain: Temperature**	14	49.0	1.39	0.1465	171	25.6	2e-16 ***

Data were obtained from 3 independent experiments, each with 3 Petri dishes, (***) represent p-value less than 0.001.

Assessment of *in-vitro* sporulation also showed a significant effect of strain and temperature as well as a significant interaction strain x temperature as confirmed by ANOVA (**Table 1**). Again, LS means grouping showed that except at 28°C where no distinct difference between strains can be observed, the Iranian isolates are all in a group distinct from the French strain, with 25°C as the best temperature for all (**Figure 2C**). However, in contrast to hyphal growth where the French isolate had higher growth rates than the Iranian ones, sporulation of the Iranian isolates was superior to that of the French strain.

#### 3.1.1 Phenotypic evaluation of *Medicago truncatula* response to an Iranian isolate points to quantitative resistance and reveals similar geographical correlation as with a French isolate

Given the homogenous growth and sporulation rates among the Iranian isolates, isolate AF1 was retained for the following study.

Ten-day-old *M. truncatula* plants were root-inoculated with spores of *V. alfalfae* isolate AF1 and symptoms were scored regularly for four weeks. Appearance of symptoms was observed 7–10 days after inoculation in susceptible *M. truncatula* lines, highly susceptible lines reaching the ultimate score of 4 (dead plant) after 3 weeks. A wide range of variation in the response, typical for quantitative traits, was observed among the 242 *M. truncatula* accessions, as shown by Area Under Disease Progress Curve (AUDPC) and Maximum Symptom Score (MSS) values (**Supplementary Figure 2**, **Supplementary Tables 5**, **6**).

Broad sense heritability (H2) values for AUDPC and MSS were 0.719 and 0.724 respectively; the correlation between AUDPC and MSS was 0.96.

These results show that the population and the phenotype scoring method are suitable to implement a genome-wide association study in order to investigate the genetic architecture of *M. truncatula* response towards Iranian *V. alfalfae*.

**Figure 3:**
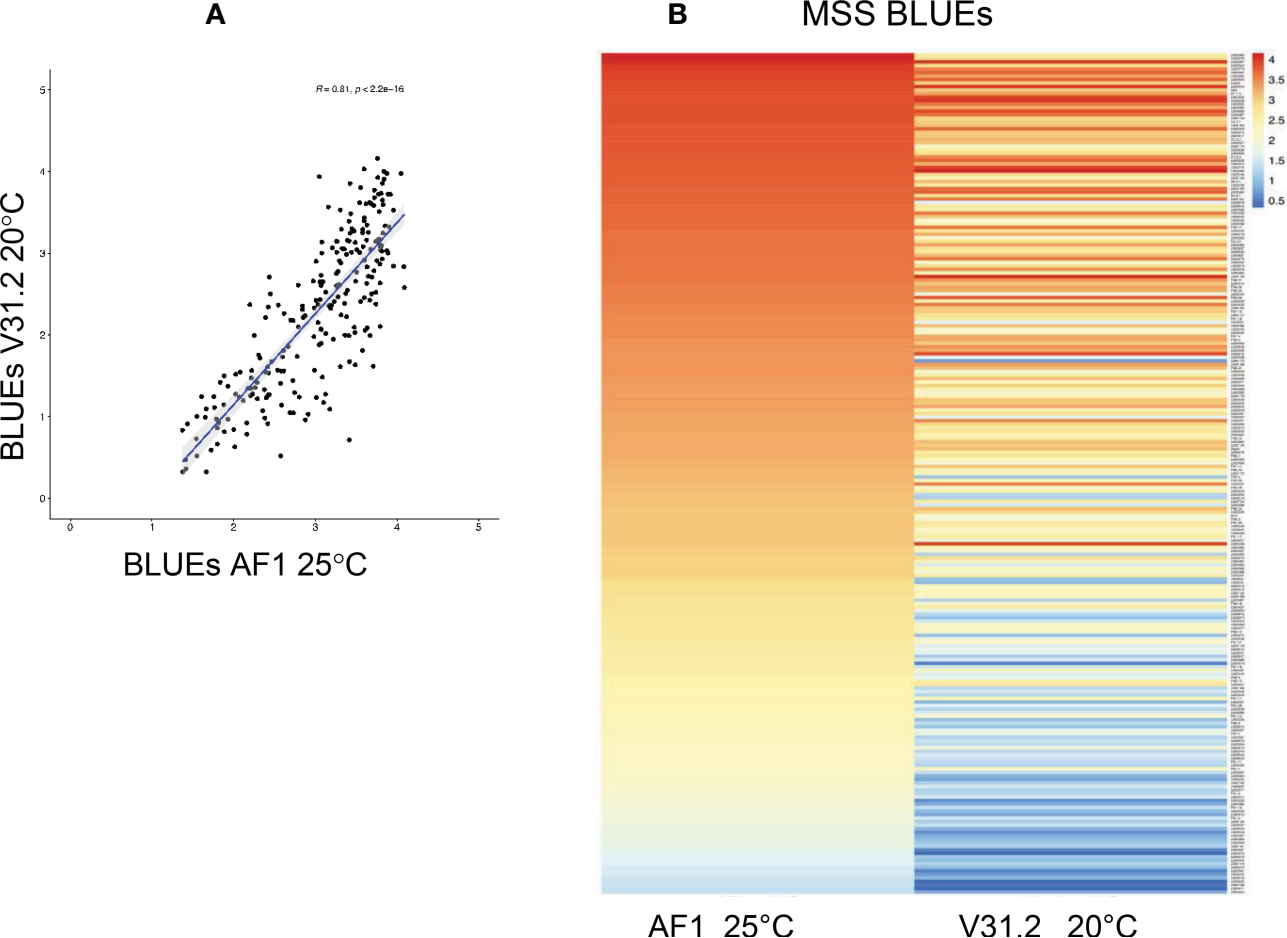
Comparison of the Maximum Symptom Score (MSS) response of a panel of 242 *M. truncatula* accessions inoculated with *Verticillium alfalfae* strain AF-1 at 25 °C and strain V31.2 at 20 °C. **(A)** Pearson correlation coefficient. **(B)** Heatmap of MSS response.

When the results of our study were compared to those of a previous one with French isolate V31.2 at 20°C, the Pearson coefficient shows a high correlation between the two studies *(*R = 0,81, p < 2,2e-16) (**Figure 3A**). It also appeared that the plants’ response in the current study had a tendency towards higher values of disease parameters, *i.e.* plants were more susceptible (**Figure 3B**). Some accessions presented a different response between the two studies. Only 5 accessions were strictly resistant to AF1 at 25°C with MSS values lower than 1.5, as compared to 63 accessions in the study with V31.2 at 20°C. Among these 63 accessions 16 became truly susceptible when inoculated with AF1 at 25°C, with MSS values ≥ 2.5.

**Figure 4:**
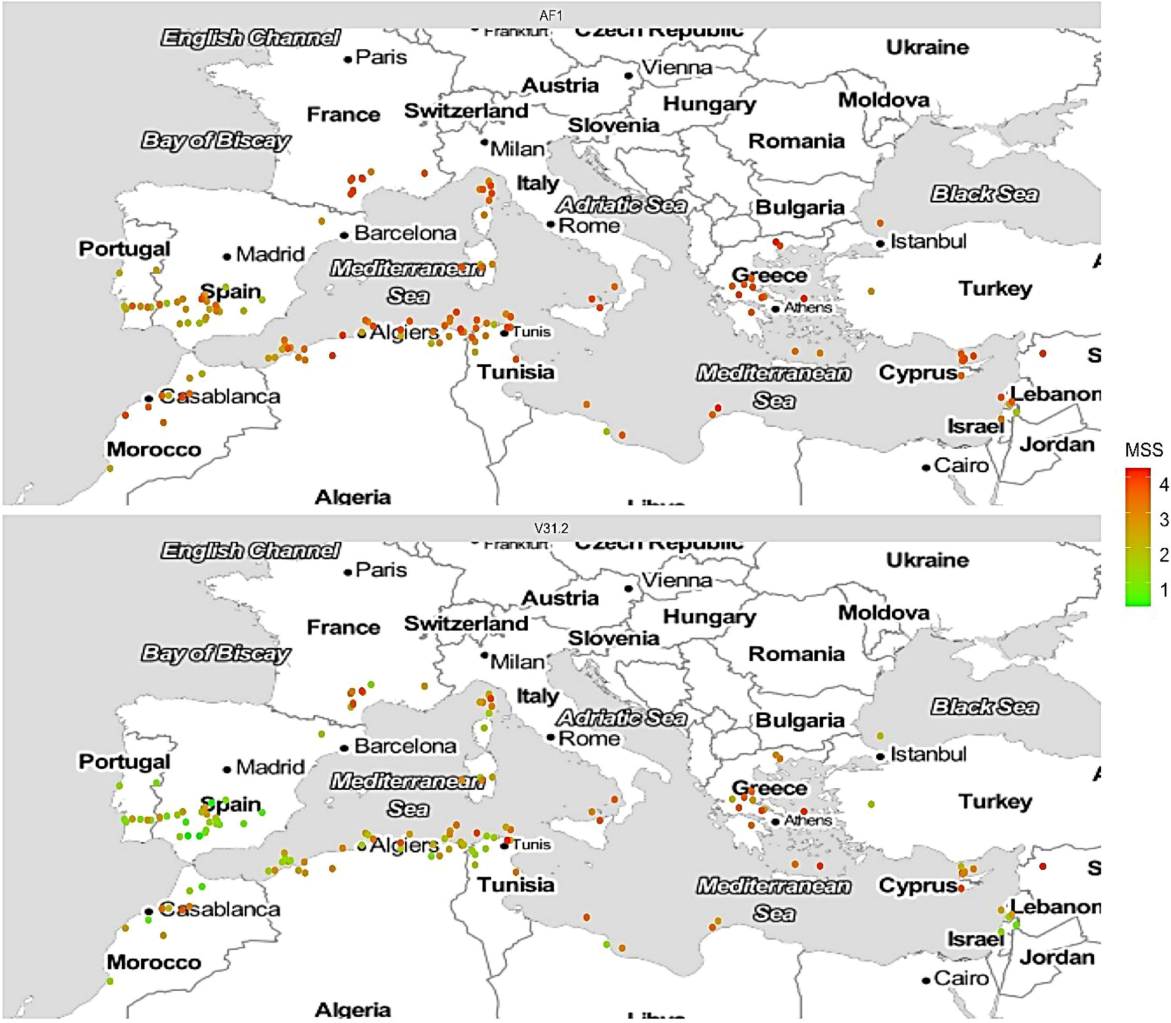
Comparison of the Maximum Symptom Score (MSS) response of a panel of 242 *M. truncatula* accessions inoculated with *Verticillium alfalfae* strain AF1 at 25°C and strain V31.2 at 20°C, across their natural geographical origins. Geographical documented origin of the 242 accessions of *M. truncatula* for which the response to the two strains of *V. alfalfae* has been evaluated is plotted on the map. Each accession is represented by a dot whose color varies according to the corrected MSS after inoculation by AF1 (upper panel) and V31.2 (lower panel). The color scale is shown on the right side, red is for susceptible, green for resistant response.

When geographical origin and response to AF1 were plotted, a gradient from east to west of susceptible and resistant accessions was evidenced, similar to the previous study with the French isolate V31.2 at 20°C (**Figure 4**).

#### 3.1.2 Genome-wide association study reveals the presence of numerous *loci* linked to resistance towards *Verticillium alfalfae* isolate AF1 in *Medicago truncatula*

**Figure 5:**
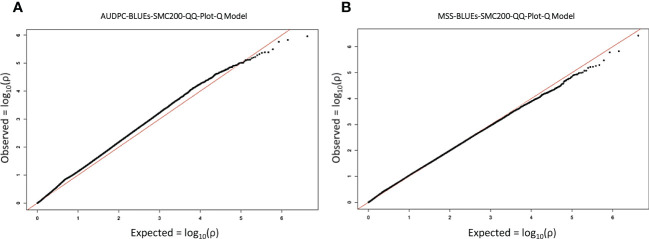
QQ plots of the MLM Q-model extracted from GWAS results. **(A)** AUDPC, **(B)** MSS. Each point represents the observed value of the P-value as a function of its theoretical value for an SNP, the more the points follow the bisector (red line), the more efficient the model. MLM, Mixed Linear Model. BLUE, the Best Linear Unbiased estimator. SMC200 refers to the site minimum count in the tassel program that was set to the 200.

In order to detect *loci* related to *M. truncatula* resistance against the Iranian isolate AF1, a genome-wide association study was undertaken using 5,671,743 high density SNP markers from the *Medicago truncatula* HapMap project.Statistical models for the association of genotype and phenotype were tested with the disease parameters MSS and AUDPC using the Tassel 5 software as in a previous study (Mazurier, 2018), and compared by Q-Q plot. As judged by this analysis, the most suitable model was a MLM Q-model which accounts for population structure for both traits, and uses the identity matrix to model kinship among random effects (**Figure 5**, **Supplementary Figure 3**). The K and Q + K models of MLMs showed deflation of p-values suggesting overfitting (**Supplementary Figure 3G–J**).

**Figure 6:**
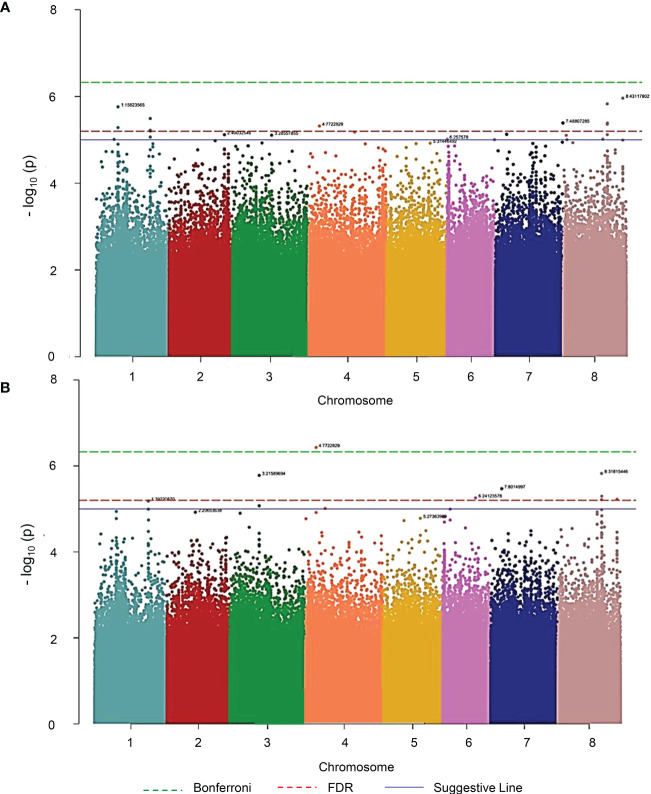
Manhattan plot illustration of the response of *M. truncatula* to *V. alfalfae* strain AF1 revealed by GWAS analysis. **(A)** AUDPC, **(B)** MSS. The green dashed line represents the Bonferroni threshold, the red dashed line the FDR threshold and the blue solid line the suggestive line of the qqman R package. AUDPC and MSS phenotypes were evaluated in 242 root-inoculated accessions of the MtHapMap collection. Association genetic analysis was performed using a Mixed linear model - Q Model. Each point represents a SNP. The X-axis shows the 8 chromosomes ordered by number, the Y-axis represents the association score, i.e. the probability that the SNP is related to the studied phenotype, is calculated as -log10[p-value].

Analysis of the association between SNPs and AUDPC (**Figure 6A**) and SNPs and MSS (**Figure 6B**) revealed a high number of strongly associated *loci*. After conservative correction for multiple testing based on SNPs association score >= 5 as determined by the suggestive line, 24 and 14 significant SNPs were identified respectively for the AUDPC and MSS parameters on seven and six chromosomes respectively (**Table 2**). Among these significant SNPs, eight SNPs were common to AUDPC and MSS, with chromosome 8 containing five significant common SNPs. Chromosomes 1, 4 and 7 contain only one common SNP each.

**Table 2:** Most significant Single Nucleotide Polymorphism (SNP) markers associated to the response of *Medicago truncatula* to *Verticillium alfalfae* strain AF1.

Trait	SNP marker	Chr	Position	df	p	MarkerR2	score	Allele	Effect
AUDPC	1:12964012	1	12964012	2	9,742E-06	0,092	5,011	A/G	0,007/0,004
AUDPC	1:15800708	1	15800708	2	5,228E-06	0,105	5,282	A/G	-0,009/-0,005
AUDPC	1:15823565	1	15823565	2	1,732E-06	0,112	5,761	C/T	-0,005/0,001
**AUDPC**	**1:39220870**	**1**	**39220870**	**2**	**3,220E-06**	**0,097**	**5,492**	**A/G**	**-0,008/-0,004**
AUDPC	1:39222678	1	39222678	2	6,012E-06	0,097	5,221	A/T	0,002/-0,002
AUDPC	1:39226123	1	39226123	2	8,624E-06	0,089	5,064	A/G	-0,008/-0,004
AUDPC	1:39333665	1	39333665	2	6,292E-06	0,090	5,201	A/T	0,004/-0,0001
AUDPC	2:33302078	2	33302078	2	4,598E-06	0,098	5,337	A/T	0,001/0,007
AUDPC	2:40032546	2	40032546	2	7,623E-06	0,087	5,118	C/T	0,003/-0,001
AUDPC	3:28557855	3	28557855	2	7,810E-06	0,092	5,107	C/T	-0,006/-0,003
**AUDPC**	**4:7722829**	**4**	**7722829**	**2**	**4,808E-06**	**0,099**	**5,318**	**G/T**	**-0,005/0,0004**
AUDPC	4:33221889	4	33221889	2	6,654E-06	0,090	5,177	A/G	0,001/0,009
AUDPC	6:257578	6	257578	2	9,656E-06	0,101	5,015	A/T	0,002/-0,005
AUDPC	6:34475434	6	34475434	2	9,889E-06	0,092	5,005	A/G	0,011/0,002
**AUDPC**	**7:8014997**	**7**	**8014997**	**2**	**7,468E-06**	**0,093**	**5,127**	**A/T**	**-0,01/-0,006**
AUDPC	7:48807285	7	48807285	2	4,102E-06	0,111	5,387	A/C	-0,005/0,001
AUDPC	8:2106964	8	2106964	2	7,865E-06	0,090	5,104	G/T	-0,008/-0,004
AUDPC	8:2597013	8	2597013	2	9,890E-06	0,098	5,005	C/T	-0,008/-0,003
**AUDPC**	**8:28608045**	**8**	**28608045**	**2**	**9,622E-06**	**0,095**	**5,017**	**C/G**	**-0,01/-0,006**
**AUDPC**	**8:31815446**	**8**	**31815446**	**2**	**1,482E-06**	**0,105**	**5,829**	**A/G**	**-0,0003/-0,007**
AUDPC	8:31823520	8	31823520	2	4,349E-06	0,094	5,362	A/G	0,004/-0,001
**AUDPC**	**8:31847916**	**8**	**31847916**	**2**	**7,590E-06**	**0,117**	**5,120**	**C/T**	**-0,0002/-0,007**
**AUDPC**	**8:31944993**	**8**	**31944993**	**2**	**4,112E-06**	**0,095**	**5,386**	**A/G**	**-0,0003/-0,005**
**AUDPC**	**8:43117802**	**8**	**43117802**	**2**	**1,094E-06**	**0,104**	**5,961**	**C/T**	**0,006/-0,003**
**MSS**	**1:39220870**	**1**	**39220870**	**2**	**6,572E-06**	**0,089**	**5,182**	**A/G**	**-6,218/-2,906**
MSS	3:21589694	3	21589694	2	1,660E-06	0,110	5,780	G/T	1,83/5,333
MSS	3:21589926	3	21589926	2	8,462E-06	0,085	5,073	C/T	-4,586/-1,384
MSS	3:21589932	3	21589932	2	8,462E-06	0,085	5,073	C/T	-1,384/-4,586
**MSS**	**4:7722829**	**4**	**7722829**	**2**	**3,732E-07**	**0,118**	**6,428**	**G/T**	**-4,438/0,674**
MSS	4:14324686	4	14324686	2	9,742E-06	0,095	5,011	G/T	4,156/-1,281
MSS	6:24123578	6	24123578	2	5,540E-06	0,088	5,257	A/G	-1,457/1,346
**MSS**	**7:8014997**	**7**	**8014997**	**2**	**3,388E-06**	**0,098**	**5,470**	**A/T**	**-8,927/-5,172**
**MSS**	**8:28608045**	**8**	**28608045**	**2**	**9,870E-06**	**0,092**	**5,006**	**C/G**	**-7,034/-3,649**
MSS	8:28608060	8	28608060	2	8,712E-06	0,094	5,060	A/T	1,653/-1,706
**MSS**	**8:31815446**	**8**	**31815446**	**2**	**1,496E-06**	**0,102**	**5,825**	**A/G**	**-0,384/-6,424**
**MSS**	**8:31847916**	**8**	**31847916**	**2**	**6,065E-06**	**0,114**	**5,217**	**C/T**	**-0,319/-6,458**
**MSS**	**8:31944993**	**8**	**31944993**	**2**	**5,069E-06**	**0,091**	**5,295**	**A/G**	**-0,403/-4,639**
**MSS**	**8:43117802**	**8**	**43117802**	**2**	**5,934E-06**	**0,088**	**5,227**	**C/T**	**4,966/-2,522**

Significant SNPs in common between AUDPC and MSS are highlighted in bold.

SNPs were filtered by p-value using the MLM Q-Model for AUDPC and MSS traits in response to inoculation with AF1.

### 3.2 Expression of selected candidate genes is induced by inoculation and differs in resistant and susceptible plants

To identify genes underlying the resistance-related *loci* revealed by GWAS, annotated genes in a span of 10 Kb upstream and downstream of each significant SNP were analyzed.

In a first step, 79 and 43 putative candidate genes were identified with the parameters AUDPC and MSS respectively. To reduce these numbers for expression studies, genes whose functional annotation suggested involvement in resistance, either by defense mechanisms or signaling pathways alongside with genes with high association score and encoding hypothetical proteins, were retained. This resulted in 52 and 27 candidate genes for AUDPC and MSS respectively. In the next step only genes that were common for AUDPC and MSS were considered which led to 21 candidate genes. Among them nine genes were chosen for expression studies (**Supplementary Table 3**), based on the availability of efficient primers in qRT-PCR.

They encode proteins such as Rho-like GTP-binding protein (Medtr8g075240), casein kinase I-like protein (Medtr1g042280), MATH domain protein (Medtr1g042160), proteasome subunit alpha type-7-A protein (Medtr8g075320), osmosensor histidine kinase (Medtr8g075340), 3-hydroxyisobutyrate dehydrogenase-like 1**/**6-phosphogluconate dehydrogenase NAD-binding domain protein (Medtr8g102470), glycoside hydrolase family 1 protein (Medtr4g023000), pathogenesis-related thaumatin family protein (Medtr8g075550), and finally a hypothetical protein without known function (Medtr1g087500).

Their expression was studied in roots inoculated with spores of *V. alfalfae* AF1 versus control condition, at early (0, 4, and 24 hpi) and intermediate (96 hpi) time points. In order to erase individual differences due to the genetic background, pools of the most resistant and the most susceptible accessions were used for RNA extraction (**Supplementary Table 4**).

**Figure 7:**
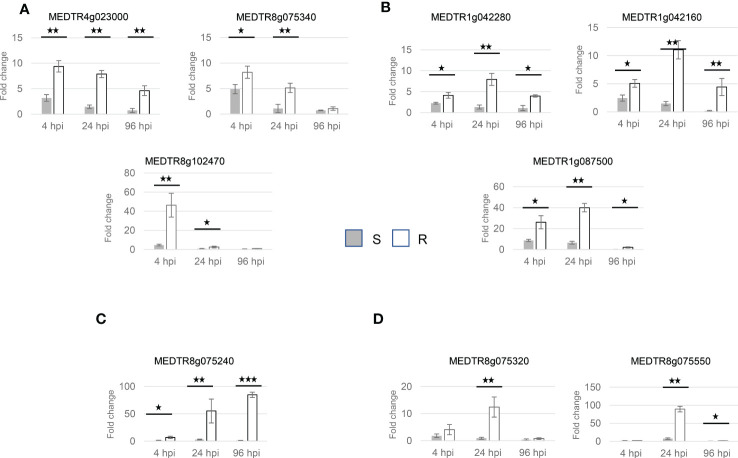
Gene expression in roots inoculated with *V. alfalfae* strain AFI, grouped by expression patterns. **(A)** Glycoside hydrolase family 1 protein (MEDTR4g023000), Osmosensor histidine kinase (MEDTR8g075340), 6-phosphogluconate dehydrogenase NAD-binding domain protein/3-hydroxyisobutyrate dehydrogenase-like 1 (MEDTR8g102470). **(B)** Casein kinase I-like protein (MEDTR1g042280), MATH domain protein (MEDTR1g042160), hypothetical protein MTR_1g087500 (MEDTR1g087500). **(C)** Rho-like GTP-binding protein (MEDTR8g075240). **(D)** Proteasome subunit alpha type-7-A protein (MEDTR8g075320), Pathogenesis-related thaumatin family protein (MEDTR8g075550). The fold change (expression in inoculated roots over expression in mock-inoculated roots) is calculated through ΔΔ CT by 2^-(ΔΔCT)^. The error bars represent the standard deviation of the fold-change. The fold change is calculated through ΔΔ CT by 2^-(ΔΔCT)^. The stars present significance levels derived from the T-test of mean comparison between treated susceptible and resistant pools at each time point. (*) represent p-value less than 0.05, (**) less than 0.01, (***) less than 0.001.

Expression analysis by qRT-PCR showed that all selected genes were expressed in roots of the susceptible and resistant plants (**Figure 7**). Moreover, their expression was induced transiently by inoculation and induction was generally stronger in plants of the resistant pool with induction factors up to 89 fold for gene Medtr8g075550 (**Figure 7D**).

Based on the time course of their expression the genes were classified into four groups (**Figure 7**). The first group contains the genes Medtr4g023000 (glycoside hydrolase family 1 protein), Medtr8g075340 (osmosensor histidine kinase) and Medtr8g102470 (3-hydroxyisobutyrate dehydrogenase-like 1**/**6-phosphogluconate dehydrogenase NAD-binding domain protein). They showed a very early induction at 4 hpi in both susceptible and resistant plants which decreased thereafter. Expression returned to basic levels in susceptible plants but stayed at induced levels for at least 24h in the resistant plants (**Figure 7A**).

The second group contains the genes Medtr1g042280 (casein kinase I-like protein), Medtr1g042160 (MATH domain protein) and Medtr1g087500 (hypothetical protein). They also exhibited very early induction at 4 hpi in both susceptible and resistant plants which was higher in resistant plants, but compared to genes of the first group their maximum expression was at 24 hpi and their induction lasted until 96 hpi (**Figure 7B**).

The third group contains only one gene, Medtr8g075240 (Rho-like GTP-binding protein), and exhibited induction at 4 hpi in resistant plants which increased until 96 hpi whereas in susceptible plants only a weak induction at 24h was observed (**Figure 7C**).

The fourth group containing the genes Medtr8g075320 (proteasome subunit alpha type-7-A protein) and Medtr8g075550 (pathogenesis-related thaumatin family protein) exhibited strong induction at 24 hpi which decreased thereafter in resistant plants. In susceptible plants the expression was induced weakly only for Medtr8g075550, at 24h and even slightly suppressed for both genes at 96hpi (**Figure 7D**).

Taken together, the expression patterns of all candidate genes detected under *loci* identified by GWAS support the involvement of these *loci* in the resistance response to *V. alfalfae*. Although all selected candidate genes were induced to some level in both susceptible and resistant plants, the difference between the resistant and susceptible plants was statistically significant at most time points, with induction factors consistently higher in the resistant plants (Pešić et al., 2014).

## 4 Discussion

The first report of Verticillium wilt disease due *V. alfalfae* in Iran was published in 2004 (Ghalandar et al., 2004) describing the pathogen in alfalfa fields of the Markazi Province. Fourteen years after this publication, the samples we collected from different parts of Iran reveal that this disease has spread since then to various areas of the Markazi and neighboring Hamedan provinces and up to the Teheran region. Since alfalfa culture in Iran depends on irrigation, it can be supposed that the pathogen spread has been favoured by irrigation systems, as has been reported for *V. dahliae* on olive tree (Jiménez-Díaz et al., 2011; López-Escudero and Mercado-Blanco, 2011). Seed companies in Iran and neighboring countries should thus include partial resistance to Verticillium wilt in future alfalfa breeding programs and survey alfalfa producing regions for occurrence of this disease in production fields.

By hyphal growth and sporulation all Iranian isolates are in one statistical group as opposed to the French isolate. A negative correlation between hyphal growth and sporulation as observed in our study has been reported earlier for other fungal species such as *Aspergillus Niger* (Muller, 1956), *V. agaricinum* and *Schizosaccharomyces pombe* (McKoy and Trinci, 1987) and *Ascochyta rabiei* (Mahiout et al., 2015).

Together with the observation that mycelia of Iranian strains develop black pigmentation after prolonged growth on PDA whereas the French strain does not, this indicates strongly that *V. alfalfae* in Iran belongs to a different genotype. An Iranian strain could thus be used to study the disease response of *M. truncatula* in a perspective of putative invasion of new pathogen strains through global trade. Also, in order to take into account a predicted increase in future temperatures, root-inoculated plants were maintained at 25°C instead of the standard temperature of 20°C which is the standard temperature for the assessment of commercial alfalfa varieties and was used in previous works (Ben et al., 2013; Toueni et al., 2016). The here described work should thus lead to detecting putatively new *loci* involved in the genetic control of resistance compared to previous studies (Ben et al., 2013; Toueni et al., 2016; Mazurier, 2018), in a scenario of globalization and global warming.

Despite the fact that two parameters have been changed (temperature and strain), there was still a high correlation between the current study at 25°C and the previous one at 20°C (Mazurier, 2018), but with a tendency towards higher susceptibility of plants towards AF1 at 25°C. This tendency could be due to the higher aggressiveness of AF1, or to the higher temperature or to a combination of both. Previous work has indeed shown that *V. alfalfae* V31.2 was more aggressive at 25°C compared to 20°C (Sbeiti et al., 2023). Studies on Fusarium wilt on two different host plants have also reported higher disease severity at higher temperature (Ferrocino et al., 2013; Chitarra et al., 2015). However, the observation that a small number of accessions changed from resistant to susceptible under the new conditions might also indicate the combined effects of strain, temperature and genotype.

The geographical distribution of susceptible and resistant accessions was similar to that revealed by the WhoGEM approach, *i.e.* resistant accessions are located in western regions of the Mediterranean basin while the susceptible ones are located in the eastern parts (Gentzbittel et al., 2019). The present results confirmed that resistance to *V. alfalfae* among *M. truncatula* accessions is structured by genome admixture and geographical origin, in a similar way independent of the pathogen’s geographical origin (**Figure 4**). However, our knowledge about Verticillium wilt in Iran is limited. Although our observations and results suggest that the Iranian *V. alfalfae* isolates belong to a different genotype than the French strain, we do not know if the pathogen is indigenous to Iran or not. It would be interesting to enlarge the study to isolates from other countries of the Mediterranean basin, east and west.

The continuous distribution of AUDPC and MSS scores through the panel of *M. truncatula* accessions indicates that resistance to the Iranian strain of *V. alfalfae* is controlled in a polygenic manner. The broad sense heritability (H^2^) calculated values for both traits (AUDPC=0.719, MSS=0.724) suggests that their variability is linked to a combination of genetic and environmental factors with a higher contribution of genetic variance.

Taken together, the range of phenotypic variation of the plants’ response to the pathogen and high heritability indicated that population and traits are suitable for studying the genetic architecture of *M. truncatula* resistance towards the Iranian *V. alfalfae* isolate through genome-wide association mapping.

GWAS evaluates the statistical significance of the association between quantitative differences of a particular phenotype and specific genetic polymorphisms in a set of genetically distinct individuals (Ogura and Busch, 2015). A first step of GWAS was to select an appropriate statistical model which reduces false positives and copes with spurious associations due to population structure (Balding, 2006; Yu and Buckler, 2006; Wang et al., 2012) and population admixture (Chen et al., 2014; Gay et al., 2020) as much as possible. Based on these criteria we selected a MLM-Q Model.

A previous study (Ben et al., 2013) has reported a major QTL controlling resistance to *V. alfalfae* strain V31.2 on chromosome 7 as well as QTLs on chromosomes 2 and 6. The major QTL on chromosome 7 was later confirmed by GWAS through co-localisation of 11 and 4 different SNPs respectively related to AUDPC and MSS (Mazurier, 2018). Our present study with an Iranian strain and conducted at higher temperature (25°C *versus* 20°C) revealed a higher number of *loci* and did not show overlap with the Mazurier study (2018) except for the *locus* on chromosome 1 with a common candidate gene *Medtr1g042160* encoding a MATH domain protein. A comparison between the two studies also showed that five resistant accessions in the previous study (Mazurier, 2018), [L000411, L000513 and L000443 originating from Spain, L000620 originating from France and A17 which is predicted to originate from Spain (Gentzbittel et al., 2019)] are still categorized as resistant in our work. However, we do not know the genetic basis of the resistance in these particular accessions. It might be based on the same *loci* or might be the result of different QTLs/genes/proteins that finally led to the same phenotypic outcome.

Taken together, our results show that a simple shift in temperature combined with a new pathogen strain drastically changes the architecture of genetic control of resistance to the pathogen and notably does not involve the strong QTL on chromosome 7 which is effective against strain V31.2 at 20°C (Ben et al., 2013; Mazurier, 2018).

The high synteny between *M. truncatula* and alfalfa makes it possible to use markers from one species in the other one, as has been shown for several genes involved in the symbiosis with Rhizobium (Zhu et al., 2005). A study on resistance to Verticillium wilt in alfalfa reported significant SNP markers on five chromosomes (Zhang et al., 2014) and suggested that those on chromosome 2 and 7 might have similar locations as QTLs identified in *M. truncatula* by Ben et al. (2013). Comparing the SNPs identified in our work and those of Zhang and co-workers (2014), the positions of markers 4:14324686 and 8:28608045 indicate additional similar locations on the alfalfa chromosomes, supporting the idea that results obtained in *M. truncatula* can be used to improve cultivated crops. A GWAS by Yu et al. (2017) described ten SNPs associated to resistance to *V. alfalfae*, some of them on the same chromosomes as in our study. However they were not near the SNPs we detected. All nine genes for which we studied expression in *M. truncatula* roots, have homologs in alfalfa (https://medicago.legumeinfo.org/tools/sequenceserver/), with more than 90% identity, though the coverage was low for three among them.

In order to validate the *loci* detected by GWAS, we selected genes in the areas 10 kb upstream and downstream of all significant SNPs. This distance is based on data on linkage disequilibrium and recombination rates described for *M. truncatula* in a previous study (Branca et al., 2011).

Chromosome 8 shows the highest number of genes putatively involved in resistance towards AF1 with 31 and 17 genes for AUDPC and MSS respectively.

One gene (Medtr1g042160) on chromosome 1 which was detected through AUDPC was common to the previous study with the French strain at 20°C (Mazurier, 2018). This gene encodes a MATH domain and coiled-coil domain-containing protein homologous to At3g58250. Meprin and TRAF-C Homology (MATH) domain is a protein-protein interaction domain composed of seven anti-parallel p-helices (Zapata et al., 2007) which is reported to play a role in plant–fungal interactions (Oelmuller et al., 2005) and also in plant responses to abiotic stress in Arabidopsis and rice as well as to pathogen attack in rice (Kushwaha et al., 2016). In our study its expression was induced by inoculation in roots at 4 hpi, to a higher level in resistant plants compared to susceptible ones. This candidate gene that was detected by two independent GWAS in response to two different strains of *V. alfalfae* and temperatures is a very promising breeding target to improve *Verticillium* resistance in Medics. Further analyses will be implemented to validate its role in the interaction between *Medicago* sp. and *Verticillium* and maybe develop molecular markers for marker-assisted selection.

In addition to the MATH gene, expression of eight other genes from *loci* on chromosomes 1, 4, 7 and 8 was studied in roots of susceptible and resistant plants after root inoculation.

These genes encode proteins mainly related to signaling and defense in stress resistance.

Genes encoding a casein kinase 1 like protein, an osmosensor histidine kinase, and a Rho-like GTP binding protein were induced at 4 hpi in *M. truncatula* roots inoculated with AF1, to a higher level in resistant plants compared to susceptible ones. Kinases through phosphorylation of their targets participate in many signaling pathways, as do GTP-binding proteins, and have putative roles in phytohormone signaling and defense against biotic stress (Pham et al., 2012; Yin et al., 2015; Klessig et al., 2018; Li et al., 2019; Zhao et al., 2020).

The gene encoding a proteasome subunit alpha type-7-A protein was induced at 4 hpi, to a higher level in resistant plants compared to susceptible ones. Ubiquitination is also an important part of signaling pathways in response to pathogens (Marino et al., 2012).

Other proteins participate more directly to defense such as the pathogenesis-related proteins to which belong thaumatin and glycoside hydrolases (Ali et al., 2018). The induction of the gene encoding the thaumatin family protein was later than that of the eight other genes, which is consistent with a role as defense protein, whereas the gene encoding glycoside hydrolase family 1 protein had its highest induction at 4hpi which indicates a possible signaling involvement of this protein.

Finally, the genes encoding a 6-phosphogluconate dehydrogenase NAD-binding domain protein/probable 3-hydroxyisobutyrate dehydrogenase-like 1 protein and a hypothetical protein, are representatives of primary metabolism and unknown functions. They were also induced in roots by AF1 inoculation, early and stronger in resistant plants, indicating their putative involvement in the plants’ resistance response.

Taken together, the expression patterns of these genes, *i.e.* strong induction in resistant plants vs. weak induction in susceptible plants, are in agreement with the claim that the *loci* identified by GWAS have significant contributions to resistance. The evaluation of the response of the association panel to the French *Verticillium* isolate V31.2 at 20°C in a previous study led to the identification of 34 candidate genes (Mazurier, 2018). Our present study with the Iranian *Verticillium* isolate AF1 at 25°C identified 93 candidate genes, with only one gene (Medtr1g042160) in common, which corresponds to about 1% overlap between the two studies. Due to the high number of candidate genes and the small contribution of each *locus* to resistance, it did not seem reasonable to go further for functional studies.

In addition, since two variables (temperature and pathogen strain) have been changed, it is not possible to separate their effects on the outcome of the interaction between *M. truncatula* and *V. alfalfae.* However, the results show that such combined effects can completely change the genetic architecture of plant disease resistance and should be a warning to breeders and institutions that control exchange of plant material.

## Data availability statement

The raw data supporting the conclusions of this article will be made available by the authors, without undue reservation.

## Author contributions

AF performed all experiments, analyzed the data and wrote a first draft of the manuscript. CB contributed substantially to the data analysis and interpretation and critically revised the manuscript. AE contributed to collecting the samples and acquisition of funding, and revised the manuscript. MM provided phenotypic data and interpretation of data. MG contributed to collecting and identifying the samples. LG contributed substantially to the conception and design of the work and supervised statistical analysis and interpretation of data. MR conceived and supervised the project, contributed to acquire funding, and revised the manuscript. All authors contributed to the article and approved the submitted version.
