# Supplementary material for: Medicago truncatula quantitative resistance to a new strain of Verticillium alfalfae from Iran revealed by a genome-wide association study
Source: Front Plant Sci. 2023 Apr 14;14:1125551. doi: 10.3389/fpls.2023.1125551 (PMC10140629; doi:10.3389/fpls.2023.1125551)
Supplement: Supplementary file 1 [file DataSheet_1.pdf]

Supplementary Material

Medicago truncatula quantitative resistance to a new strain of *Verticillium alfalfae* from Iran revealed by a Genome-wide association study

Amir H. Fartash[1], Cécile Ben[1,2] Mélanie Mazurier[1], Asa Ebrahimi[3], Mojtaba Ghalandar[4], Laurent Gentzbittel [1,2], Martina Rickauer[1]

*** Correspondence:**
Martina Rickauer
martina.rickauer@toulouse-inp.fr

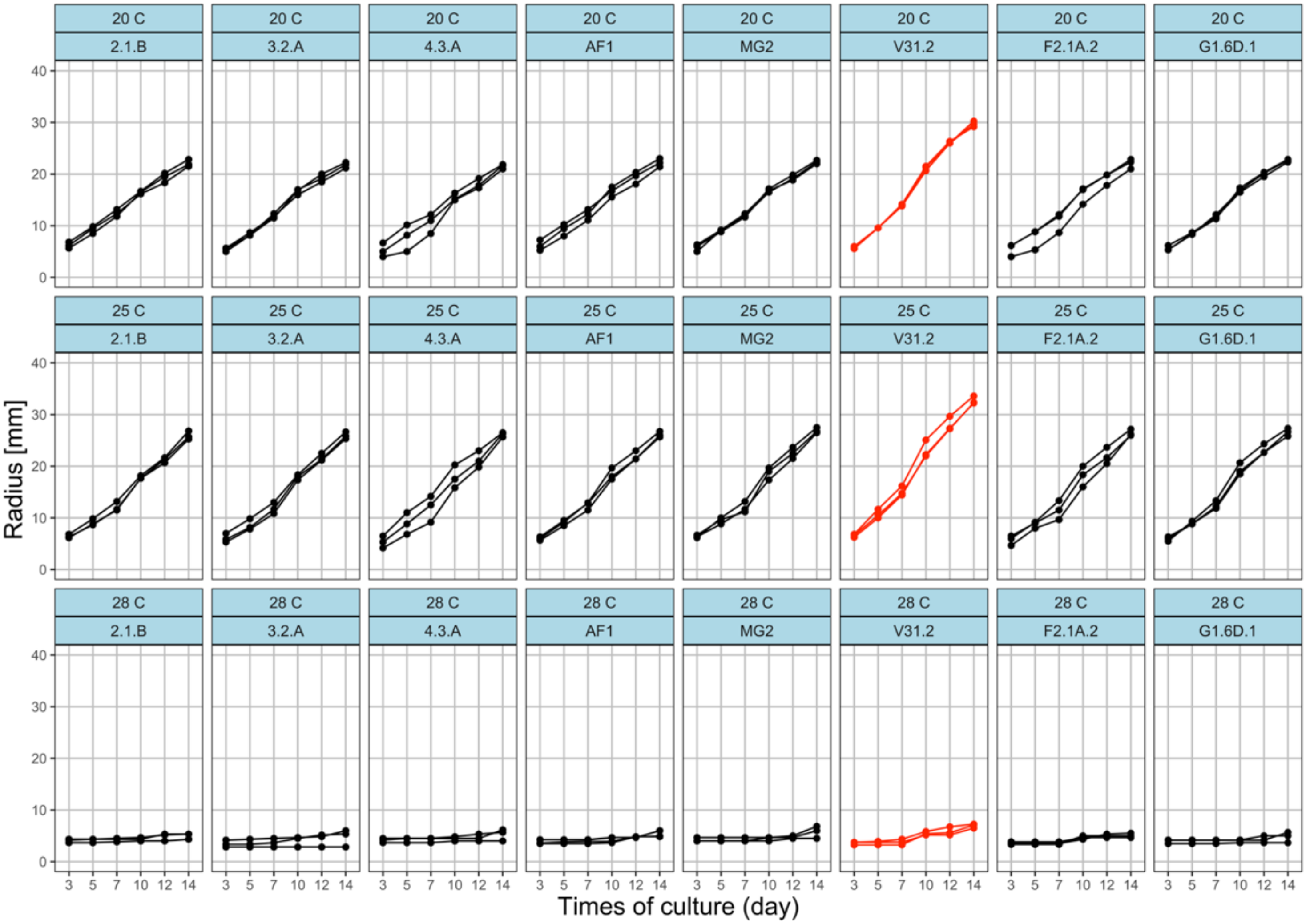

Supplementary Figure 1. Radial growth of Iranian and French strains at three temperatures.

Fungal samples were cultured on PDA in darkness. The growth was measured as radius during 14 days at specified intervals. The red curves represent the French isolate. Curves show mean values of 3 independent experiments, each with 3 Petri dishes.

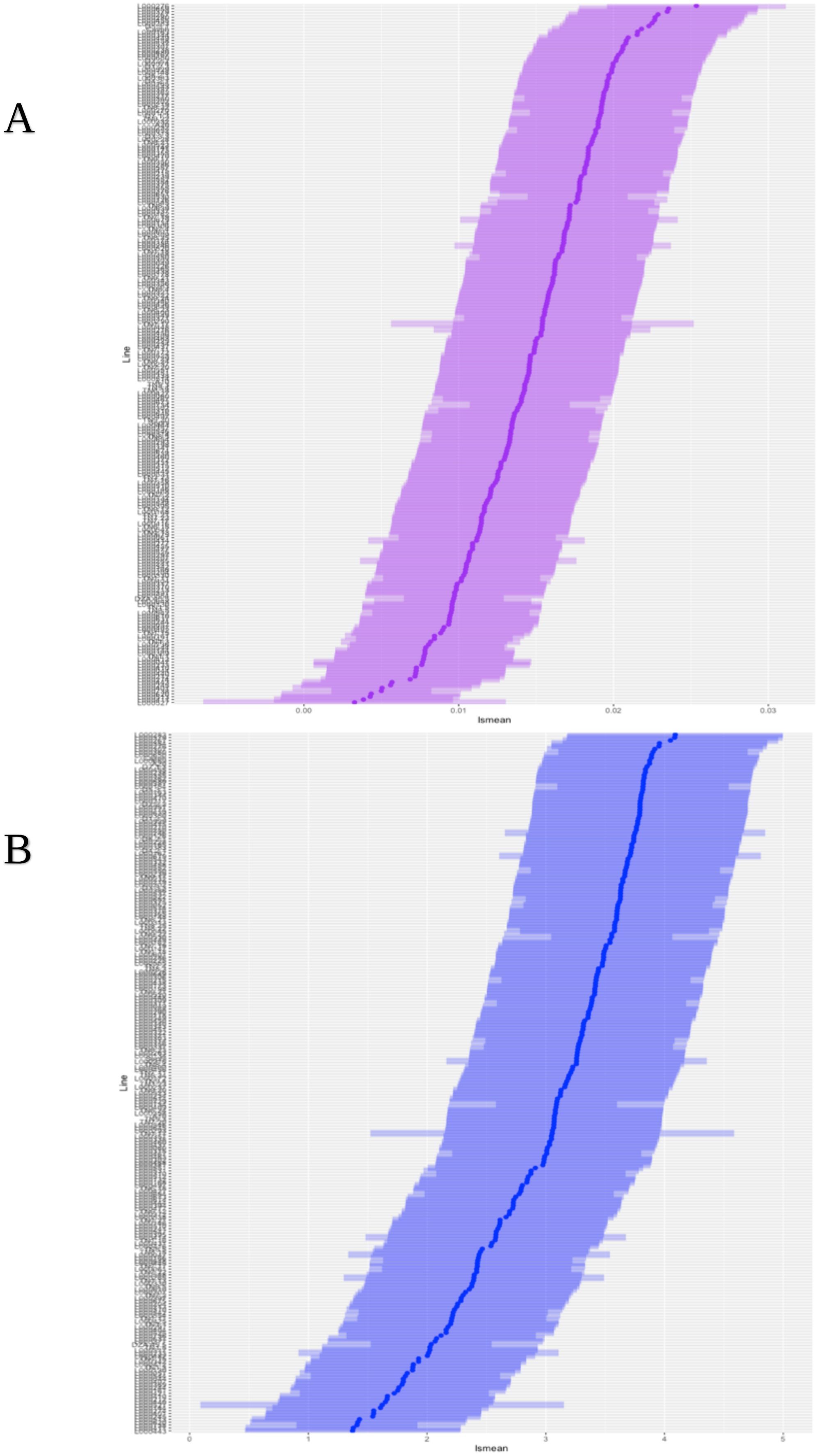

Supplementary Figure 2. Response variation to inoculation with *V.* alfalfae AF-1 within the *M. truncatula* association panel for AUDPC and MSS.
A: LSmeans value of AUDPC. B: LSmeans value of MSS. The values are arranged in ascending order. The high number of accessions makes the x-axis difficult to read. For detailed information refer to supplementary tables 5 and 6.

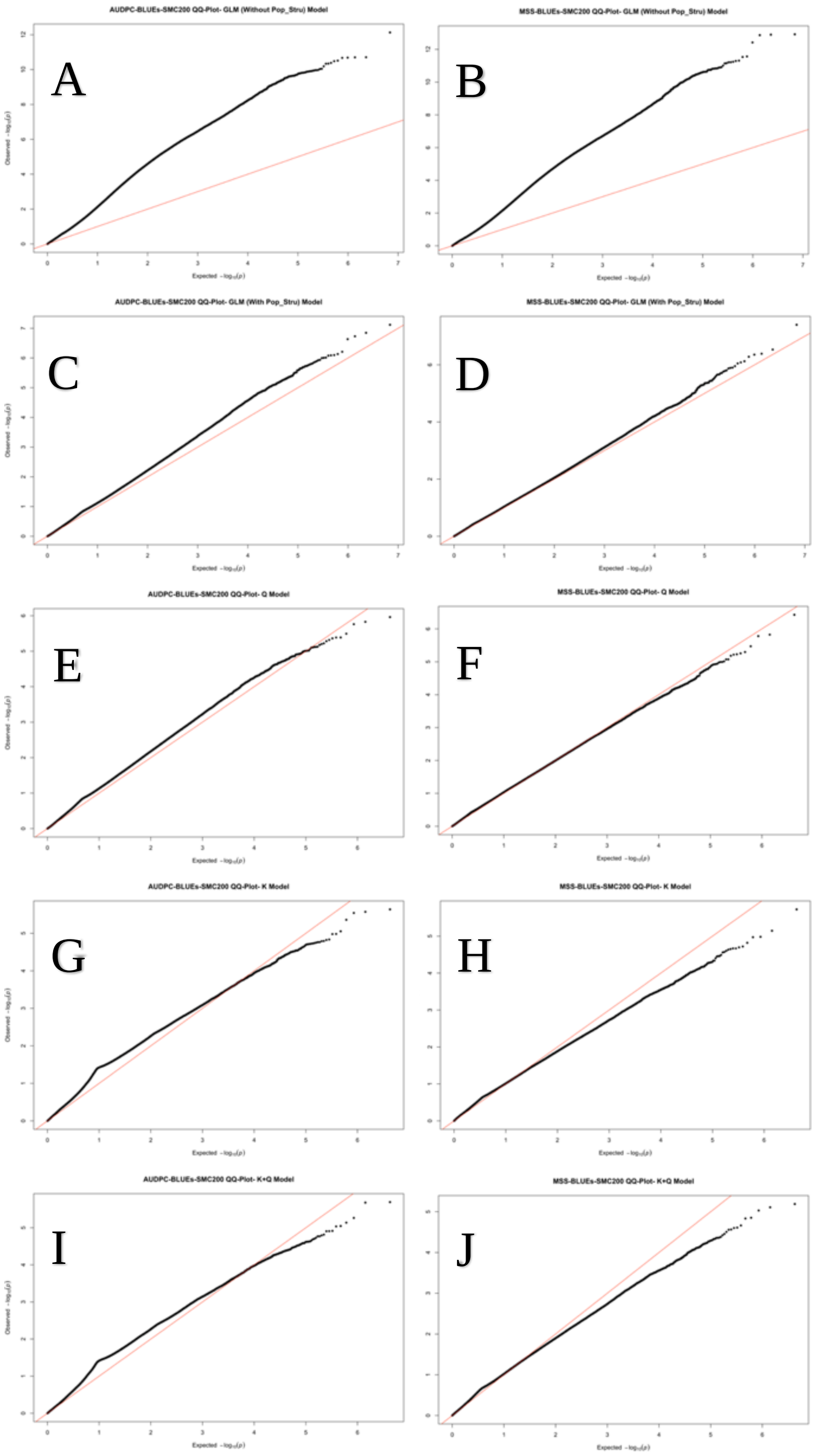

Supplementary Figure 3. Q-Q plots of different models of GWAS for AUDPC (A, C, E, G, H) and MSS (B, D, F, H, J). A, B: naïve GLM model, test of SNP/trait association with no correction for population structure, C, D: GLM Q-model using population structure as fixed covariate, E, F: MLM Q-Model; a mixed-effects model including population structure as a fixed effect and identity matrix for kinship as random effect, G, H: MLM- K-Model, a mixed model including the SNP-based kinship matrix among genotypes as a random effect, but no population structure as fixed effect, I, H: Q+K-model including population structure as a fixed effect and SNP-based kinship matrix among genotypes for random effects.

Supplementary Table 1. Primers used for molecular identification of selected fungal samples

Specificity	Primer name	Direction	Sequence (5' -> 3')	Annealing Temperature (°C)	
Fungal ITS	ITS1	Forward	TCCGTAGGTGAACCTGCGG	50 *	(White et al., 1990)
	ITS4	Reverse	TCCTCCGCTTATTGATATGC		
V. alfalfae	AlfF	Forward	TCATGCCCCCTTTGTTCATCGAT	62*	(Inderbitzin et al., 2013)
	AlfD1r	Reverse	TGCCGGCATCGACCTTGG		
V. nonalfalfae	NoF	Forward	CCTCGAAAAATCCACCAGCTCTA	65*	
	NoNuR	Reverse	GTGGTTGAGATCCTCACGCTTC		

Supplementary Table 2. *Medicago truncatula* accessions used in this study. The table is extracted from the Medicago HapMap project (https://medicagohapmap2.org/germplasm), * CC refers to the INRA *Medicago truncatula* core collection (https://www1.montpellier.inra.fr/BRC-MTR/mauguio/mauguio.php?page=menu15&numero=1), RIL = recombinant inbred lines

	ID	Line	Population	Country	Category *
**1**	HM001	L000163	SA022322	Syria	CC8
**2**	HM002	L000174	SA028064	Cyprus	CC8
**3**	HM003	L000544	ESP 105-L	Spain	CC8
**4**	HM004	L000736	DZA45.6	Algeria	CC8
**5**	HM005	L000734	DZA315.16	Algeria	CC8
**6**	HM006	L000530	F83005.5	France	CC8
**7**	HM007	L000651	SALSES 71B	France	CC8
**8**	HM008	L000368	DZA 012-J	Algeria	CC8
**9**	HM009	L000555	GRC 020-B	Greece	CC16
**10**	HM010	L000154	SA024714	Italy	CC16
**11**	HM011	L000543	DZA 327-7	Algeria	CC16
**12**	HM012	L000239	SA026063	Morocco	CC16
**13**	HM013	L000648	SALSES 42B	France	CC16
**14**	HM014	L000542	DZA 233-4	Algeria	CC16
**15**	HM015	L000550	F 11013-3	France	CC16
**16**	HM016	L000049	SA009707	Tunisia	CC16
**17**	HM017	A10	A10	Unknown	RIL.Parent
**18**	HM018	A20	A20	Unknown	RIL.Parent
**19**	HM019	L000527	Borung	Unknown	RIL.Parent
**20**	HM020	TN1.11	TN1.11	Tunisia	RIL.Parent
**21**	HM021	TN1.21	TN1.21	Tunisia	RIL.Parent
**22**	HM022	TN3.23	TN3.23	Tunisia	RIL.Parent
**23**	HM023	TN6.18	TN6.18	Tunisia	RIL.Parent
**24**	HM024	TN8.3	TN8.3	Tunisia	RIL.Parent
**25**	HM025	TN9.22	TN9.22	Tunisia	RIL.Parent
**26**	HM026	L000450	F 11008-C	France	RIL.Parent/CC96
**27**	HM027	L000531	F83005-9	France	RIL.Parent/CC144
**28**	HM028	L000342	DZA241-2	Algeria	RIL.Parent/CC144

	ID	Line	Population	Country	Category *
**29**	HM029	L000729	R108.C3	Unknown	M. tricycla
**30**	HM031	L000545	ESP 158-A	Spain	CC32
**31**	HM032	L000549	F 11005-E	France	CC32
**32**	HM033	L000552	F 20047-A	France.Corsica	CC32
**33**	HM034	L000554	F 20089-B	France.Corsica	CC32
**34**	HM035	L000679	F 66017	France	CC32
**35**	HM036	L000337	GRC 042-1	Greece	CC32
**36**	HM037	L000557	GRC 064-B	Greece	CC32
**37**	HM038	L000369	PRT 180-A	Portugal	CC32
**38**	HM039	L000263	SA003116	Israel	CC32
**39**	HM040	L000321	SA003780	Italy	CC32
**40**	HM041	L000198	SA009048	Libya	CC32
**41**	HM042	L000290	SA009119	Turkey	CC32
**42**	HM043	L000310	SA009944	Tunisia	CC32
**43**	HM044	L000245	SA014161	Jordan	CC32
**44**	HM045	L000144	SA014163	Jordan	CC32
**45**	HM046	L000213	SA027882	Morocco	CC32
**46**	HM047	L000654	DZA 014	Algeria	CC64
**47**	HM048	L000475	DZA 016-F	Algeria	CC64
**48**	HM049	L000360	DZA 058-5	Algeria	CC64
**49**	HM050	L000477	DZA 058-J	Algeria	CC64
**50**	HM051	L000357	DZA 202-4	Algeria	CC64
**51**	HM052	L000639	DZA 210-3	Algeria	CC64
**52**	HM053	L000601	DZA 246-6	Algeria	CC64
**53**	HM054	L000395	DZA 309-A	Algeria	CC64
**54**	HM055	L000675	DZA 326	Algeria	CC64
**55**	HM056	L000416	ESP 074-A	Spain	CC64
**56**	HM057	L000512	ESP 155-D	Spain	CC64
**57**	HM058	L000513	ESP 163-E	Spain	CC64
**58**	HM059	L000449	F 11012-A	France	CC64
**59**	HM060	L000520	F 20015-10	France.Corsica	CC64
**60**	HM061	L000645	GRC 033-B2	Greece	CC64
**61**	HM062	L000379	GRC 063-D	Greece	CC64
**62**	HM063	L000525	PRT 176-F	Portugal	CC64
**63**	HM064	L000371	PRT 178-D	Portugal	CC64

	ID	Line	Population	Country	Category *
**64**	HM065	L000370	PRT 179-J	Portugal	CC64
**65**	HM066	L000204	SA001489	Israel	CC64
**66**	HM067	L000265	SA001526	Algeria	CC64
**67**	HM068	L000274	SA003648	Portugal	CC64
**68**	HM069	L000276	SA007749	Tunisia	CC64
**69**	HM070	L000307	SA008625	Morocco	CC64
**70**	HM071	L000297	SA008626	Morocco	CC64
**71**	HM072	L000061	SA009357	Algeria	CC64
**72**	HM073	L000052	SA009710	Tunisia	CC64
**73**	HM074	L000314	SA009866	Algeria	CC64
**74**	HM075	L000130	SA012451	Italy	CC64
**75**	HM076	L000234	SA023859	Tunisia	CC64
**76**	HM077	L000232	SA027062	Greece	CC64
**77**	HM078	L000228	SA027063	Greece	CC64
**78**	HM079	L000362	DZA 045-4-C	Algeria	CC96
**79**	HM080	L000637	DZA 061-B3d	Algeria	CC96
**80**	HM081	L000356	DZA 202-5	Algeria	CC96
**81**	HM082	L000355	DZA 210-2	Algeria	CC96
**82**	HM083	L000354	DZA 210-5	Algeria	CC96
**83**	HM084	L000394	DZA 243-6	Algeria	CC96
**84**	HM085	L000673	DZA 319	Algeria	CC96
**85**	HM086	L000674	DZA 322	Algeria	CC96
**86**	HM087	L000397	DZA 323-1	Algeria	CC96
**87**	HM088	L000497	DZA 323-D	Algeria	CC96
**88**	HM089	L000431	ESP 155-A	Spain	CC96
**89**	HM090	L000437	ESP 163-A	Spain	CC96
**90**	HM091	L000548	ESP 171-F	Spain	CC96
**91**	HM092	L000443	ESP 174-A	Spain	CC96
**92**	HM093	L000444	ESP 173-A	Spain	CC96
**93**	HM094	L000574	F 13006-1	France	CC96
**94**	HM095	L000458	F 20025-4	France.Corsica	CC96
**95**	HM096	L000460	F 20026-F	France.Corsica	CC96
**96**	HM097	L000522	F 83005-G	France	CC96
**97**	HM098	L000523	PRT 176-12	Portugal	CC96

	ID	Line	Population	Country	Category *
**98**	HM099	L000202	SA002840	Cyprus	CC96
**99**	HM101	L000738	A17_Varma	Unknown	Unknown
**100**	HM103	L000302	SA004087	Tunisia	CC96
**101**	HM104	L000303	SA008604	Algeria	CC96
**102**	HM105	L000293	SA009137	Algeria	CC96
**103**	HM106	L000060	SA009434	Tunisia	CC96
**104**	HM107	L000047	SA009670	Algeria	CC96
**105**	HM108	L000317	SA009715	Tunisia	CC96
**106**	HM109	L000126	SA011959	Israel	CC96
**107**	HM110	L000225	SA021560	Libya	CC96
**108**	HM111	L000219	SA027192	Italy	CC96
**109**	HM112	L000178	SA028097	Cyprus	CC96
**110**	HM113	L000537	CRE007-J	Greece. Crete	CC144
**111**	HM114	L000338	CRE009-A	Greece. Crete	CC144
**112**	HM115	L000529	Cyprus_C	Cyprus	CC144
**113**	HM116	L000659	DZA211	Algeria	CC144
**114**	HM117	L000401	ESP031-A	Spain	CC144
**115**	HM118	L000410	ESP043-B	Spain	CC144
**116**	HM119	L000414	ESP050-B	Spain	CC144
**117**	HM120	L000482	ESP095-C	Spain	CC144
**118**	HM122	L000546	Esp159-11	Spain	CC144
**119**	HM123	L000440	ESP163-B	Spain	CC144
**120**	HM124	L000547	Esp165-D	Spain	CC144
**121**	HM125	L000445	ESP175-A	Spain	CC144
**122**	HM126	L000456	F20015-L	France.Corsica	CC144
**123**	HM127	L000455	F20025-F	France.Corsica	CC144
**124**	HM128	L000620	F20058-6	France.Corsica	CC144
**125**	HM129	L000468	F20069-A	France.Corsica	CC144
**126**	HM130	L000467	F20069-C	France.Corsica	CC144
**127**	HM131	L000551	F34042-D	France	CC144
**128**	HM132	L000376	GRC093-C	Greece	CC144
**129**	HM133	L000375	GRC098-A	Greece	CC144
**130**	HM134	L000267	SA02084	Greece	CC144
**131**	HM135	L000332	SA02748	Israel	CC144
**132**	HM136	L000270	SA02820	Turkey	CC144

	ID	Line	Population	Country	Category *
**133**	HM137	L000322	SA03749	Israel	CC144
**134**	HM138	L000283	SA08454	Libya	CC144
**135**	HM139	L000306	SA08623	Morocco	CC144
**136**	HM140	L000286	SA09049	Libya	CC144
**137**	HM141	L000044	SA09456	Tunisia	CC144
**138**	HM142	L000315	SA09820	Libya	CC144
**139**	HM143	L000132	SA10481	Tunisia	CC144
**140**	HM144	L000241	SA15951	Portugal.Madeira	CC144
**141**	HM145	L000146	SA19964	Turkey	CC144
**142**	HM146	L000237	SA21302	Libya	CC144
**143**	HM147	L000161	SA21362	Libya	CC144
**144**	HM148	L000162	SA21590	Libya	CC144
**145**	HM149	L000148	SA21819	Cyprus	CC144
**146**	HM150	L000238	SA22323	Syria	CC144
**147**	HM151	L000165	SA25226	Italy	CC144
**148**	HM152	L000166	SA25654	Morocco	CC144
**149**	HM153	L000168	SA25898	Italy	CC144
**150**	HM154	L000173	SA27778	Morocco	CC144
**151**	HM155	L000216	SA27961	Morocco	CC144
**152**	HM156	L000217	SA28089	Cyprus	CC144
**153**	HM157	L000215	SA28099	Cyprus	CC144
**154**	HM158	L000649	Salse46B	France	CC144
**155**	HM159	L000680	arboretu	France	CC192
**156**	HM160	L000340	CRE005-A	Greece. Crete	CC192
**157**	HM161	L000365	DZA033-2	Algeria	CC192
**158**	HM162	L000538	DZA055-H	Algeria	CC192
**159**	HM163	L000358	DZA061-11	Algeria	CC192
**160**	HM164	L000350	DZA215-5	Algeria	CC192
**161**	HM165	L000344	DZA231-1	Algeria	CC192
**162**	HM166	L000343	DZA236-2	Algeria	CC192
**163**	HM167	L000341	DZA242-A	Algeria	CC192
**164**	HM168	L000400	DZA323-3	Algeria	CC192
**165**	HM169	L000404	ESP039-A	Spain	CC192
**166**	HM170	L000407	ESP042-B	Spain	CC192

	ID	Line	Population	Country	Category *
**167**	HM171	L000409	ESP045-A	Spain	CC192
**168**	HM172	L000412	ESP048-E	Spain	CC192
**169**	HM173	L000411	ESP048-F	Spain	CC192
**170**	HM174	L000610	ESP095-9	Spain	CC192
**171**	HM175	L000510	ESP098-B	Spain	CC192
**172**	HM176	L000421	ESP098-C	Spain	CC192
**173**	HM177	L000425	ESP100-G	Spain	CC192
**174**	HM178	L000427	ESP103-B	Spain	CC192
**175**	HM179	L000438	ESP162-A	Spain	CC192
**176**	HM180	L000514	ESP163-C	Spain	CC192
**177**	HM181	L000448	ESP175-D	Spain	CC192
**178**	HM182	L000451	F11013-A	France	CC192
**179**	HM184	L000463	F20058-B	France.Corsica	CC192
**180**	HM185	L000470	F20081-A	France.Corsica	CC192
**181**	HM186	L000387	GRC024-H	Greece	CC192
**182**	HM187	L000386	GRC033-C	Greece	CC192
**183**	HM188	L000383	GRC040-1	Greece	CC192
**184**	HM189	L000372	PRT177-C	Portugal	CC192
**185**	HM190	L000330	SA02806	Portugal	CC192
**186**	HM191	L000277	SA03653	Portugal	CC192
**187**	HM192	L000280	SA07763	Tunisia	CC192
**188**	HM193	L000309	SA08638	Morocco	CC192
**189**	HM194	L000057	SA09700	Tunisia	CC192
**190**	HM195	L000313	SA09728	Tunisia	CC192
**191**	HM196	L000207	SA09970	Tunisia	CC192
**192**	HM197	L000246	SA12455	Italy	CC192
**193**	HM198	L000244	SA18543	Tunisia	CC192
**194**	HM199	L000147	SA19983	Cyprus	CC192
**195**	HM200	L000134	SA19998	Cyprus	CC192
**196**	HM201	L000233	SA24576	Morocco	CC192
**197**	HM202	L000226	SA25941	Italy	CC192
**198**	HM203	L000167	SA27176	Greece	CC192
**199**	HM204	L000172	SA27185	Italy	CC192
**200**	HM205	L000212	SA28375	Portugal	CC192
**201**	HM206	L000650	Salse57A	France	CC192

	ID	Line	Population	Country	Category *
**202**	HM207	Caliph	Caliph	Unknown	Unknown
**203**	HM209	Sephi	Sephi	Unknown	Unknown
**204**	HM210	TN1.1	TN1.1	Tunisia	Tunisian.lines
**205**	HM211	TN1.17	TN1.17	Tunisia	Tunisian.lines
**206**	HM212	TN1.3	TN1.3	Tunisia	Tunisian.lines
**207**	HM213	TN7.11	TN7.11	Tunisia	Tunisian.lines
**208**	HM215	TN7.17	TN7.17	Tunisia	Tunisian.lines
**209**	HM217	TN7.19	TN7.19	Tunisia	Tunisian.lines
**210**	HM218	TN7.2	TN7.2	Tunisia	Tunisian.lines
**211**	HM219	TN7.20	TN7.20	Tunisia	Tunisian.lines
**212**	HM220	TN7.22	TN7.22	Tunisia	Tunisian.lines
**213**	HM222	TN7.4	TN7.4	Tunisia	Tunisian.lines
**214**	HM223	TN8.15	TN8.15	Tunisia	Tunisian.lines
**215**	HM224	TN8.21	TN8.21	Tunisia	Tunisian.lines
**216**	HM225	TN8.22	TN8.22	Tunisia	Tunisian.lines
**217**	HM226	TN8.23	TN8.23	Tunisia	Tunisian.lines
**218**	HM227	TN8.24	TN8.24	Tunisia	Tunisian.lines
**219**	HM228	TN8.25	TN8.25	Tunisia	Tunisian.lines
**220**	HM230	TN8.4	TN8.4	Tunisia	Tunisian.lines
**221**	HM231	TN8.5	TN8.5	Tunisia	Tunisian.lines
**222**	HM232	TN9.12	TN9.12	Tunisia	Tunisian.lines
**223**	HM234	TN9.17	TN9.17	Tunisia	Tunisian.lines
**224**	HM235	TN9.20	TN9.20	Tunisia	Tunisian.lines
**225**	HM236	TN9.21	TN9.21	Tunisia	Tunisian.lines
**226**	HM237	TN9.24	TN9.24	Tunisia	Tunisian.lines
**227**	HM238	TN9.5	TN9.5	Tunisia	Tunisian.lines
**228**	HM239	TN1.5	TN1.5	Tunisia	Tunisian.lines
**229**	HM240	TN1.13	TN1.13	Tunisia	Tunisian.lines
**230**	HM241	TN1.15	TN1.15	Tunisia	Tunisian.lines
**231**	HM242	TN1.16	TN1.16	Tunisia	Tunisian.lines
**232**	HM243	TN1.18	TN1.18	Tunisia	Tunisian.lines
**233**	HM244	TN9.3	TN9.3	Tunisia	Tunisian.lines
**234**	HM245	TN9.4	TN9.4	Tunisia	Tunisian.lines
**235**	HM280	D1.2.3	D1.2.3	Syria	Damas lines

	ID	Line	Population	Country	Category *
**236**	HM281	D2.2.2	D2.2.2	Syria	Damas lines
**237**	HM282	D3.3.3	D3.3.3	Syria	Damas lines
**238**	HM283	D4.2.1	D4.2.1	Syria	Damas lines
**239**	HM284	D5.3.1	D5.3.1	Syria	Damas lines
**240**	HM285	D6.2.1	D6.2.1	Syria	Damas lines
**241**	HM286	D7.1.3	D7.1.3	Syria	Damas lines
**242**	Unkown	DZA 45.5	DZA045-5	Algeria	CC8

Supplementary Table 3. Primers designed for gene expression study

Gene	Functional annotation	Direction	T_m (°C)	% Efficiency	Product Size (bp)	Sequence
Medtr1g042280	Casein kinase I-like protein	Left	59.8	89,55	127	CCTTCTTGGACCCAGTCTCG
		Right	60.2			CAGAAAACCCCGAGAGTGCA
Medtr1g042160	MATH domain protein	Left	58.7	101,21	86	ACTCGGAGCCTTACGTTCTT
		Right	59.4			TCCTAACTGGTCGACTGCAC
Medtr4g023000	Glycoside hydrolase family 1 protein	Left	60.0	100,99	135	TCTCACGCTGCAGCAGTAAA
		Right	60.0			CCGAGACGTTGCTTCTCTGT
Medtr8g075240	Rho-like GTP-binding protein	Left	59.9	90,91	82	ATGCACCTGGTGTTCCCATT
		Right	59.5			CCTGGATGGTCGACGAAGAA
Medtr8g075320	Proteasome subunit alpha type-7-A protein	Left	60.0	115,50	141	CGGTGTTCGCGGTATCGATA
		Right	59.9			GCATCAGCTTTTAGCCCAGC

Gene	Functional annotation	Direction	T_m (°C)	% Efficiency	Product Size (bp)	Sequence
Medtr8g075340	Osmosensor histidine kinase	Left	59.9	103,07	150	GGGAGATTCTGCGAGAGTGG
		Right	59.7			GGCTTCTGCTCCAGGGTAAA
Medtr8g075550	Pathogenesis-related thaumatin family protein	Left	59.3	101,32	150	CCACGCGCTTATAGCTATGC
		Right	59.8			TGCCCAAATGTCCACTCCAA
Medtr8g102470	6-phosphogluconate dehydrogenase NAD-binding domain protein	Left	60.1	100,96	80	TGTCACTCAATCGACGCTCC
		Right	60.0			TCTCCTCCGGCGAATATTGC
Medtr1g087500	Hypothetical protein	Left	60.1	98,43	82	TGGAATCTCCAGCAAGGTCTG
		Right	60.5			CGCAAAACCTTGAGTCGTCG

Supplementary Table 4. *Medicago truncatula* accessions used for gene expression analysis. CC refers to the INRA *Medicago truncatula* core collection (https://www1.montpellier.inra.fr/BRC-MTR/mauguio/mauguio.php?page=menu15&numero=1)

ID	Line	Origin	Category	BLUEs_AUDPC	BLUEs_MSS	Response to AF1
**HM173**	L000411	Spain	CC192	0,003847	1,378796	Resistant
**HM058**	L000513	Spain	CC64	0,004280	1,431084	Resistant
**HM128**	L000620	France.Corsica	CC144	0,004348	1,416628	Resistant
**HM101**	L000738	Unknown	Unknown	0,005001	1,409722	Resistant
**HM117**	L000401	Spain	CC144	0,005049	1,549689	Resistant
**HM044**	L000245	Jordan	CC32	0,005598	1,543006	Resistant
**HM092**	L000443	Spain	CC96	0,005690	1,372750	Resistant
**HM068**	L000274	Portugal	CC64	0,006866	1,550761	Resistant
**HM205**	L000212	Portugal	CC192	0,007169	1,662605	Resistant
**HM123**	L000440	Spain	CC144	0,007215	1,649124	Resistant
**HM141**	L000044	Tunisia	CC144	0,007248	1,800373	Resistant
**HM118**	L000410	Spain	CC144	0,007265	1,666440	Resistant
**HM059**	L000449	France	CC64	0,020947	3,541393	Susceptible
**HM165**	L000344	Algeria	CC192	0,021528	3,803844	Susceptible
**HM001**	L000163	Syria	CC8	0,021559	3,811832	Susceptible
**HM207**	Caliph	Unknown	Unknown	0,021808	3,889299	Susceptible
**HM284**	D5.3.1	Syria	Damas	0,022285	3,817523	Susceptible
**HM138**	L000283	Libya	CC144	0,022491	4,090339	Susceptible
**HM127**	L000455	France.Corsica	CC144	0,022646	3,840099	Susceptible
**HM192**	L000280	Tunisia	CC192	0,022688	3,762328	Susceptible
**HM134**	L000267	Greece	CC144	0,022874	4,050639	Susceptible
**HM062**	L000379	Greece	CC64	0,023474	4,085681	Susceptible
**HM116**	L000659	Algeria	CC144	0,023557	3,875298	Susceptible
**HM069**	L000276	Tunisia	CC64	0,025352	3,954020	Susceptible

Supplementary Table 5. Response variation to inoculation with *V. alfalfae* AF-1 within the *M. truncatula* association panel for AUDPC. The estimated AUDPC values are adjusted through Mixed Linear Model. Values are arranged in ascending order.

	Line	AUDPC Lsmean	SE	lower.CL	upper.CL
1	L000527	0,003272502	0,00261965	-0,0065	0,013045
2	L000411	0,003846547	0,00154718	-0,001931	0,009625
3	L000513	0,00428002	0,00154778	-0,0015	0,01006
4	L000620	0,004348327	0,00154889	-0,001436	0,010133
5	L000738	0,00500124	0,00084857	0,001774	0,008229
6	L000401	0,00504879	0,00154775	-0,000731	0,010829
7	L000245	0,005598412	0,00154879	-0,000186	0,011382
8	L000443	0,005689907	0,00154778	-9,03E-05	0,01147
9	L000274	0,006865962	0,001549	0,001081	0,012651
10	L000212	0,007168931	0,00154788	0,001388	0,01295
11	L000440	0,007215376	0,00154828	0,001433	0,012997
12	L000044	0,007248351	0,0015483	0,001466	0,013031
13	L000410	0,007264657	0,00154828	0,001483	0,013047
14	L000233	0,007609475	0,00187239	0,000622	0,014597
15	L000047	0,007661472	0,0018833	0,000633	0,01469
16	L000412	0,007727306	0,00154807	0,001946	0,013509

	Line	AUDPC Lsmean	SE	lower.CL	upper.CL
17	TN1.1	0,007755319	0,00155027	0,001966	0,013545
18	L000369	0,00781794	0,00155207	0,002022	0,013614
19	L000144	0,007820907	0,00155253	0,002023	0,013619
20	L000544	0,007874716	0,00135775	0,002798	0,012951
21	L000736	0,008052286	0,00135775	0,002976	0,013129
22	TN1.3	0,008184387	0,00155027	0,002395	0,013974
23	L000421	0,008427171	0,00135502	0,003361	0,013493
24	L000161	0,008437119	0,00154879	0,002653	0,014221
25	TN1.15	0,008832162	0,00154935	0,003046	0,014618
26	L000482	0,008959995	0,00154828	0,003178	0,014742
27	L000407	0,00905363	0,00154774	0,003274	0,014834
28	L000057	0,009348936	0,00154788	0,003568	0,01513
29	L000244	0,009380469	0,00154802	0,003599	0,015162
30	L000637	0,009406259	0,00155253	0,003608	0,015204
31	L000610	0,00943206	0,00154733	0,003654	0,015211
32	L000542	0,009495267	0,00135775	0,004419	0,014572
33	TN9.5	0,009545285	0,00155047	0,003755	0,015336
34	TN1.5	0,009559241	0,00154976	0,003772	0,015347
35	L000130	0,00957275	0,00155259	0,003774	0,015371
36	L000648	0,009613909	0,00135775	0,004538	0,01469

	Line	AUDPC Lsmean	SE	lower.CL	upper.CL
37	DZA 45.5	0,0096813	0,00084857	0,006454	0,012909
38	L000554	0,009726862	0,00154778	0,003947	0,015507
39	L000397	0,009737429	0,00154775	0,003957	0,015518
40	L000213	0,009836903	0,00154781	0,004057	0,015617
41	L000370	0,009844186	0,00155259	0,004046	0,015643
42	L000317	0,009903674	0,00155106	0,004111	0,015696
43	L000404	0,01015551	0,00154836	0,004373	0,015938
44	TN1.11	0,010189849	0,00135628	0,005119	0,015261
45	L000330	0,010318511	0,00154774	0,004538	0,016099
46	L000204	0,010415565	0,001549	0,004631	0,0162
47	L000166	0,010441315	0,0015483	0,004659	0,016224
48	L000342	0,010487589	0,00154852	0,004705	0,016271
49	L000241	0,01050616	0,00154852	0,004723	0,016289
50	L000265	0,010614379	0,00187321	0,003624	0,017605
51	L000207	0,010658722	0,0015485	0,004876	0,016442
52	L000297	0,010674488	0,00154781	0,004894	0,016455
53	L000525	0,010730614	0,0015485	0,004948	0,016514
54	L000477	0,010887496	0,0015476	0,005108	0,016667
55	L000425	0,010893592	0,00154802	0,005112	0,016675
56	L000277	0,010896884	0,00154774	0,005117	0,016677

	Line	AUDPC Lsmean	SE	lower.CL	upper.CL
57	L000414	0,011150603	0,00187374	0,004158	0,018143
58	L000061	0,011194136	0,00135772	0,006118	0,01627
59	L000675	0,011268425	0,00155275	0,005469	0,017067
60	TN1.13	0,011346232	0,00155047	0,005556	0,017137
61	L000547	0,011380624	0,00154781	0,0056	0,017161
62	TN8.15	0,011414215	0,00155027	0,005625	0,017204
63	L000416	0,011459704	0,0015476	0,00568	0,017239
64	TN7.17	0,011469289	0,00154976	0,005682	0,017257
65	TN7.22	0,011470169	0,00155047	0,00568	0,017261
66	TN1.21	0,011532288	0,00154698	0,005755	0,01731
67	L000168	0,011647958	0,00154879	0,005864	0,017432
68	TN9.12	0,011654336	0,00155047	0,005864	0,017445
69	L000395	0,011681805	0,00155253	0,005884	0,01748
70	L000548	0,011777098	0,00154788	0,005997	0,017558
71	L000394	0,011872542	0,00155259	0,006074	0,017671
72	L000132	0,012004628	0,0015483	0,006222	0,017787
73	TN7.2	0,012042161	0,00155027	0,006253	0,017832
74	L000468	0,012072466	0,00154892	0,006288	0,017857
75	L000146	0,012088825	0,00154811	0,006307	0,01787
76	L000510	0,012209971	0,00154774	0,00643	0,01799

	Line	AUDPC Lsmean	SE	lower.CL	upper.CL
77	L000358	0,012362871	0,00154774	0,006583	0,018143
78	TN1.16	0,012468546	0,00154935	0,006682	0,018255
79	TN3.23	0,012518954	0,00154701	0,006742	0,018296
80	L000531	0,012523714	0,00154746	0,006745	0,018303
81	L000445	0,012571829	0,00154746	0,006793	0,018351
82	L000512	0,01269461	0,0015476	0,006915	0,018474
83	L000315	0,012721008	0,00154836	0,006939	0,018503
84	L000431	0,012738	0,00154778	0,006958	0,018518
85	L000372	0,0129365	0,00154807	0,007155	0,018718
86	L000460	0,012992802	0,0015485	0,00721	0,018776
87	L000538	0,013056961	0,00154774	0,007277	0,018837
88	L000674	0,013098413	0,00155259	0,0073	0,018897
89	L000341	0,013147484	0,00154774	0,007367	0,018928
90	L000198	0,013219504	0,00155253	0,007421	0,019018
91	L000293	0,013275198	0,00154775	0,007495	0,019055
92	L000654	0,013298895	0,0013571	0,008225	0,018373
93	TN8.5	0,013320524	0,00154976	0,007533	0,019108
94	L000314	0,013357913	0,00135756	0,008282	0,018434
95	L000546	0,013362291	0,00154746	0,007583	0,019141
96	L000357	0,013429751	0,00155259	0,007631	0,019228

	Line	AUDPC Lsmean	SE	lower.CL	upper.CL
97	L000444	0,013429909	0,0015485	0,007647	0,019213
98	Sephi	0,013469207	0,00154935	0,007683	0,019255
99	TN9.20	0,013511909	0,00155027	0,007722	0,019301
100	L000497	0,013567081	0,0015485	0,007784	0,01935
101	L000645	0,013609384	0,00155253	0,007811	0,019407
102	L000310	0,013760964	0,00135771	0,008685	0,018837
103	L000302	0,013825256	0,00155275	0,008026	0,019624
104	L000734	0,013939732	0,00084857	0,010712	0,017167
105	L000673	0,01403855	0,00155275	0,00824	0,019837
106	L000467	0,014050545	0,00135649	0,008979	0,019122
107	L000060	0,014102042	0,00155275	0,008303	0,019901
108	L000427	0,014177011	0,00154802	0,008396	0,019958
109	TN6.18	0,014248202	0,00154762	0,008469	0,020028
110	TN9.4	0,014258393	0,00154935	0,008472	0,020045
111	TN9.3	0,014273638	0,00154976	0,008486	0,020061
112	A10	0,014361926	0,00154733	0,008583	0,02014
113	L000514	0,014412239	0,00154807	0,008631	0,020194
114	L000338	0,014464599	0,00154811	0,008683	0,020246
115	L000451	0,014527459	0,00154774	0,008747	0,020308
116	L000551	0,014541675	0,00154828	0,00876	0,020324

	Line	AUDPC Lsmean	SE	lower.CL	upper.CL
117	TN7.20	0,014553113	0,00155047	0,008763	0,020343
118	L000237	0,014566972	0,00154807	0,008786	0,020348
119	TN8.24	0,014602772	0,00155027	0,008813	0,020392
120	L000729	0,014614304	0,00154698	0,008837	0,020392
121	L000523	0,014636749	0,00154788	0,008856	0,020417
122	L000172	0,014861108	0,00154788	0,009081	0,020642
123	TN7.11	0,014880815	0,00155027	0,009091	0,02067
124	L000437	0,014909402	0,0015485	0,009126	0,020692
125	L000234	0,014960682	0,00154781	0,00918	0,020741
126	L000225	0,014961853	0,00154755	0,009182	0,020741
127	L000463	0,015069199	0,00154718	0,009291	0,020847
128	L000409	0,015307431	0,00154718	0,009529	0,021085
129	L000450	0,015312923	0,00154778	0,009533	0,021093
130	L000216	0,015381722	0,00187458	0,008386	0,022377
131	L000448	0,015388159	0,00154774	0,009608	0,021168
132	TN1.17	0,015404913	0,00262045	0,00563	0,02518
133	L000290	0,015418563	0,0015483	0,009636	0,021201
134	L000371	0,015426143	0,00135755	0,010351	0,020502
135	L000549	0,015520287	0,00154778	0,00974	0,021301
136	L000400	0,015551325	0,00154802	0,00977	0,021332

	Line	AUDPC Lsmean	SE	lower.CL	upper.CL
137	TN8.23	0,015571718	0,00154976	0,009784	0,021359
138	L000649	0,015634066	0,00154852	0,009851	0,021417
139	L000456	0,01568323	0,00154892	0,009899	0,021468
140	L000340	0,01572527	0,00154807	0,009944	0,021507
141	TN9.24	0,015762557	0,00154935	0,009976	0,021549
142	L000322	0,015791712	0,00154892	0,010007	0,021576
143	L000321	0,015838295	0,00155275	0,010039	0,021637
144	TN8.4	0,015909037	0,00154935	0,010123	0,021695
145	L000263	0,015981372	0,001549	0,010197	0,021766
146	L000356	0,01604876	0,00155207	0,010252	0,021845
147	L000343	0,016083122	0,00154807	0,010302	0,021864
148	TN9.21	0,016092054	0,00155047	0,010302	0,021882
149	L000178	0,016180813	0,00155253	0,010383	0,021979
150	L000438	0,016183095	0,00154774	0,010403	0,021963
151	L000365	0,016198855	0,00154802	0,010418	0,02198
152	L000226	0,016211802	0,00154788	0,010431	0,021992
153	L000049	0,016230416	0,00155253	0,010432	0,022029
154	L000303	0,016272677	0,00155275	0,010474	0,022072
155	L000550	0,016424152	0,00135737	0,011349	0,021499
156	L000368	0,016481833	0,00155253	0,010684	0,02228

	Line	AUDPC Lsmean	SE	lower.CL	upper.CL
157	TN1.18	0,016666137	0,00155047	0,010876	0,022456
158	L000520	0,016679162	0,0015476	0,0109	0,022459
159	L000246	0,016718195	0,0018732	0,009728	0,023708
160	L000386	0,016778107	0,00154774	0,010998	0,022558
161	L000174	0,01678987	0,00155259	0,010992	0,022588
162	TN8.25	0,016798633	0,00154976	0,011011	0,022586
163	L000309	0,016819853	0,00154839	0,011037	0,022602
164	L000601	0,016852804	0,00155207	0,011056	0,022649
165	TN7.4	0,016954808	0,00155027	0,011165	0,022744
166	L000306	0,017006342	0,00154811	0,011225	0,022788
167	L000134	0,017036201	0,00154788	0,011256	0,022817
168	L000679	0,017122782	0,0018833	0,010095	0,024151
169	TN7.19	0,017138211	0,00155027	0,011349	0,022928
170	L000162	0,017147722	0,00154693	0,011371	0,022925
171	L000337	0,017163287	0,00135761	0,012087	0,022239
172	L000639	0,0171645	0,00155207	0,011368	0,022961
173	TN8.3	0,017211326	0,00154701	0,011434	0,022989
174	L000545	0,017565966	0,00135543	0,012498	0,022634
175	L000126	0,01768182	0,00155275	0,011883	0,023481
176	L000530	0,017684772	0,00084857	0,014457	0,020912

	Line	AUDPC Lsmean	SE	lower.CL	upper.CL
177	L000651	0,01771857	0,00135775	0,012642	0,022795
178	L000376	0,017797442	0,00154811	0,012016	0,023579
179	L000555	0,01780134	0,00154733	0,012023	0,02358
180	L000375	0,017810081	0,00154836	0,012028	0,023592
181	L000354	0,017812926	0,00155275	0,012014	0,023612
182	L000552	0,017854927	0,00154788	0,012074	0,023636
183	L000239	0,017919404	0,00155253	0,012121	0,023718
184	L000215	0,018122966	0,00135649	0,013051	0,023195
185	L000475	0,01812774	0,0015476	0,012348	0,023907
186	L000557	0,018158396	0,00154788	0,012378	0,023939
187	L000286	0,018250861	0,00154889	0,012466	0,024035
188	L000350	0,018266593	0,00154774	0,012487	0,024047
189	TN9.17	0,018330274	0,00154976	0,012543	0,024118
190	L000470	0,018352982	0,00154802	0,012572	0,024134
191	L000355	0,018358284	0,00155207	0,012562	0,024155
192	L000173	0,018376893	0,00154879	0,012593	0,024161
193	L000147	0,018382946	0,00154788	0,012602	0,024164
194	L000522	0,018532169	0,00154778	0,012752	0,024312
195	TN8.21	0,018609283	0,00155027	0,01282	0,024399
196	L000228	0,018663512	0,00154879	0,012879	0,024448

	Line	AUDPC Lsmean	SE	lower.CL	upper.CL
197	D3.3.3	0,018746481	0,00154775	0,012966	0,024527
198	L000313	0,018841276	0,00154718	0,013063	0,024619
199	L000052	0,018845006	0,00135755	0,013769	0,023921
200	L000232	0,018998015	0,001549	0,013213	0,024783
201	A20	0,019008592	0,00154734	0,01323	0,024787
202	L000332	0,019012462	0,00154836	0,01323	0,024795
203	TN9.22	0,019060135	0,00154762	0,01328	0,02484
204	D7.1.3	0,01912191	0,00154852	0,013339	0,024905
205	L000154	0,019194812	0,00122712	0,0146	0,023789
206	L000270	0,019209902	0,00154889	0,013426	0,024994
207	TN8.22	0,01921595	0,00155027	0,013426	0,025006
208	L000219	0,019256546	0,00154781	0,013476	0,025037
209	L000202	0,019274731	0,0015483	0,013493	0,025057
210	L000360	0,019325344	0,00135761	0,01425	0,024401
211	L000217	0,019354637	0,00154852	0,013572	0,025138
212	L000362	0,019381349	0,00155207	0,013585	0,025178
213	L000387	0,01949225	0,00154777	0,013712	0,025272
214	L000543	0,019555623	0,00154733	0,013777	0,025334
215	L000165	0,019585247	0,0015483	0,013803	0,025367
216	D4.2.1	0,019596434	0,00154911	0,013811	0,025382

	Line	AUDPC Lsmean	SE	lower.CL	upper.CL
217	L000383	0,019692091	0,00154807	0,013911	0,025473
218	D6.2.1	0,019693975	0,00154911	0,013909	0,025479
219	L000148	0,019868846	0,00154828	0,014087	0,025651
220	L000529	0,019888432	0,00154828	0,014106	0,025671
221	D1.2.3	0,019955584	0,00154911	0,01417	0,025741
222	L000574	0,019970288	0,0015476	0,014191	0,02575
223	D2.2.2	0,020150112	0,00154911	0,014365	0,025935
224	L000650	0,020204341	0,00154655	0,014429	0,02598
225	L000167	0,020318678	0,00154788	0,014538	0,026099
226	L000680	0,02049722	0,00154655	0,014722	0,026273
227	L000238	0,020588867	0,00154839	0,014806	0,026371
228	L000307	0,020622605	0,00155275	0,014824	0,026422
229	L000537	0,020783714	0,00154811	0,015002	0,026565
230	L000458	0,020828757	0,0015485	0,015046	0,026612
231	L000449	0,020946606	0,00154788	0,015166	0,026727
232	L000344	0,021528199	0,00154828	0,015746	0,02731
233	L000163	0,02155872	0,00155259	0,01576	0,027357
234	Caliph	0,021807821	0,00154976	0,01602	0,027596
235	D5.3.1	0,022285452	0,00154775	0,016505	0,028066
236	L000283	0,022490945	0,00154892	0,016706	0,028275

	Line	AUDPC Lsmean	SE	lower.CL	upper.CL
237	L000455	0,022645749	0,00154777	0,016866	0,028426
238	L000280	0,022688206	0,00154718	0,01691	0,028466
239	L000267	0,022873948	0,00154828	0,017092	0,028656
240	L000379	0,023474066	0,00155275	0,017675	0,029273
241	L000659	0,023556766	0,00154852	0,017774	0,02934
242	L000276	0,025351536	0,00154781	0,019571	0,031132

Supplementary Table 6. Response variation to inoculation with *V. alfalfae* AF-1 within the *M. truncatula* association panel for MSS; The estimated MSS values are adjusted through Mixed Linear Model. Values are arranged in ascending order.

	Line	MSS Lsmean	SE	lower.CL	upper.CL
1	L000443	1,372750174	0,24286	0,465666	2,279835
2	L000411	1,378795871	0,24277	0,472064	2,285528
3	L000738	1,409722222	0,1337	0,900431	1,919013
4	L000620	1,41662796	0,24303	0,508889	2,324367
5	L000513	1,431083508	0,24286	0,523999	2,338168
6	L000245	1,543005697	0,24302	0,635324	2,450687
7	L000401	1,549689036	0,24286	0,642616	2,456762
8	L000274	1,550761219	0,24305	0,642953	2,458569
9	L000144	1,608000263	0,24361	0,698105	2,517895
10	L000527	1,622511018	0,41056	0,090966	3,154056
11	L000440	1,649123544	0,24294	0,741739	2,556508
12	L000212	1,66260483	0,24287	0,755462	2,569748
13	L000410	1,666439561	0,24294	0,759055	2,573824
14	L000421	1,723554023	0,21273	0,928025	2,519083
15	L000161	1,753699097	0,24302	0,846018	2,66138
16	L000244	1,76316651	0,2429	0,85594	2,670393
17	L000369	1,788903933	0,24353	0,87928	2,698527

	Line	MSS Lsmean	SE	lower.CL	upper.CL
18	L000407	1,799602258	0,24285	0,892539	2,706666
19	L000044	1,800373264	0,24294	0,892979	2,707768
20	L000544	1,817955038	0,21316	1,020812	2,615099
21	L000057	1,833447876	0,24287	0,926305	2,740591
22	L000130	1,877705492	0,24362	0,967772	2,787639
23	TN1.3	1,879639064	0,24325	0,971086	2,788192
24	L000412	1,884157808	0,2429	0,976901	2,791415
25	L000342	1,927791268	0,24298	1,020267	2,835316
26	TN1.15	1,928234264	0,24311	1,020219	2,836249
27	L000482	2,002561772	0,24294	1,095177	2,909946
28	L000233	2,012764643	0,29363	0,916999	3,10853
29	L000317	2,023917764	0,24338	1,114889	2,932947
30	TN1.5	2,024552418	0,24317	1,116295	2,93281
31	DZA 45.5	2,034722222	0,1337	1,525431	2,544013
32	L000277	2,068120777	0,24285	1,161057	2,975184
33	L000637	2,080222485	0,24361	1,170327	2,990118
34	L000736	2,11845107	0,21316	1,321308	2,915595
35	L000525	2,160800467	0,24297	1,253287	3,068314
36	L000404	2,161225607	0,24295	1,253796	3,068656
37	L000297	2,176242181	0,24286	1,269138	3,083347
38	TN1.1	2,198452195	0,24325	1,289899	3,107005

	Line	MSS Lsmean	SE	lower.CL	upper.CL
39	L000425	2,204003451	0,2429	1,296777	3,11123
40	TN1.11	2,208776151	0,21293	1,412503	3,005049
41	L000554	2,21580573	0,24286	1,308721	3,12289
42	L000542	2,220614531	0,21316	1,423471	3,017758
43	L000370	2,23127692	0,24362	1,321344	3,14121
44	L000213	2,250316255	0,24286	1,343212	3,157421
45	L000204	2,259094552	0,24305	1,351287	3,166902
46	L000675	2,28409389	0,24364	1,374066	3,194122
47	L000397	2,288273285	0,24285	1,381204	3,195342
48	TN7.2	2,303881488	0,24325	1,395329	3,212434
49	L000207	2,32362098	0,24297	1,416108	3,231134
50	L000610	2,355452677	0,24279	1,448627	3,262278
51	TN9.5	2,375579062	0,24328	1,466904	3,284254
52	L000330	2,380620777	0,24285	1,473557	3,287684
53	TN1.13	2,390929939	0,24328	1,482255	3,299605
54	L000265	2,394298759	0,29376	1,298048	3,49055
55	L000548	2,397453315	0,24287	1,49031	3,304596
56	TN7.22	2,417715654	0,24328	1,509041	3,32639
57	L000061	2,417805315	0,21315	1,620685	3,214925
58	TN7.17	2,42373886	0,24317	1,515481	3,331996

	Line	MSS Lsmean	SE	lower.CL	upper.CL
59	L000445	2,423993556	0,24281	1,517093	3,330894
60	L000648	2,427115285	0,21316	1,629972	3,224259
61	L000166	2,432949021	0,24294	1,525554	3,340344
62	L000047	2,438510026	0,29534	1,336304	3,540716
63	TN8.15	2,449841084	0,24325	1,541288	3,358394
64	TN8.5	2,465272809	0,24317	1,557015	3,37353
65	L000416	2,537471906	0,24283	1,630494	3,444449
66	L000431	2,545982498	0,24286	1,638898	3,453067
67	TN1.16	2,573123865	0,24311	1,665109	3,481139
68	L000414	2,578161209	0,29384	1,481599	3,674724
69	L000395	2,580222485	0,24361	1,670327	3,490118
70	L000547	2,582988213	0,24286	1,675884	3,490093
71	L000241	2,597257913	0,24298	1,689734	3,504782
72	L000510	2,608524817	0,24285	1,701461	3,515588
73	L000146	2,614211168	0,24291	1,706929	3,521493
74	TN1.21	2,615482179	0,24273	1,708862	3,522102
75	L000538	2,665342999	0,24285	1,75828	3,572406
76	L000372	2,692164616	0,2429	1,784908	3,599421
77	TN9.12	2,69623297	0,24328	1,787558	3,604908
78	L000477	2,720672964	0,24283	1,813695	3,62765
79	L000394	2,726647291	0,24362	1,816714	3,636581

	Line	MSS Lsmean	SE	lower.CL	upper.CL
80	L000444	2,727314509	0,24297	1,819801	3,634828
81	L000674	2,731124295	0,24362	1,821191	3,641058
82	L000673	2,746792302	0,24364	1,836764	3,656821
83	L000654	2,77646652	0,21306	1,97971	3,573223
84	L000427	2,789441161	0,2429	1,882214	3,696668
85	TN6.18	2,793603119	0,24284	1,886606	3,7006
86	L000497	2,803169209	0,24297	1,895656	3,710682
87	L000168	2,840227919	0,24302	1,932547	3,747909
88	L000132	2,844273808	0,24294	1,936879	3,751668
89	L000512	2,85175762	0,24283	1,94478	3,758735
90	L000310	2,87431055	0,21315	2,077191	3,67143
91	L000531	2,876506784	0,24281	1,969606	3,783407
92	L000551	2,912759907	0,24294	2,005375	3,820144
93	L000341	2,97504294	0,24285	2,06798	3,882106
94	L000468	2,982966867	0,24304	2,075206	3,890728
95	L000302	2,985681191	0,24364	2,075653	3,895709
96	L000463	2,991036408	0,24277	2,084304	3,897769
97	L000467	3,007842792	0,21296	2,21145	3,804236
98	L000315	3,008478354	0,24295	2,101048	3,915908
99	L000060	3,01425262	0,24364	2,104224	3,924281

	Line	MSS Lsmean	SE	lower.CL	upper.CL
100	L000437	3,031024687	0,24297	2,123512	3,938538
101	L000460	3,032229039	0,24297	2,124716	3,939742
102	L000338	3,047846977	0,24291	2,140565	3,955129
103	L000357	3,047943587	0,24362	2,13801	3,957877
104	TN1.17	3,055031498	0,41069	1,523013	4,58705
105	L000293	3,057122491	0,24285	2,150054	3,964191
106	L000645	3,066333596	0,24361	2,156438	3,976229
107	L000546	3,06817345	0,24281	2,161273	3,975074
108	TN7.20	3,069898193	0,24328	2,161224	3,978573
109	TN9.3	3,071022225	0,24317	2,162765	3,97928
110	A10	3,072516169	0,24279	2,165691	3,979342
111	L000358	3,080025539	0,24285	2,172962	3,987089
112	TN8.24	3,08039664	0,24325	2,171844	3,988949
113	L000409	3,086579754	0,24277	2,179847	3,993312
114	L000734	3,090277778	0,1337	2,580987	3,599569
115	L000729	3,093995251	0,24273	2,187376	4,000615
116	L000514	3,099661055	0,2429	2,192404	4,006918
117	L000234	3,123250728	0,24286	2,216146	4,030355
118	L000523	3,125358743	0,24287	2,218216	4,032502
119	TN9.20	3,129470714	0,24325	2,220918	4,038024
120	L000237	3,159951459	0,2429	2,252695	4,067208

	Line	MSS Lsmean	SE	lower.CL	upper.CL
121	TN3.23	3,167403884	0,24274	2,260771	4,074037
122	TN9.4	3,177522013	0,24311	2,269507	4,085537
123	L000172	3,188362406	0,24287	2,281219	4,095505
124	TN9.24	3,196272013	0,24311	2,288257	4,104287
125	TN7.11	3,210952195	0,24325	2,302399	4,119505
126	L000456	3,220988105	0,24304	2,313227	4,128749
127	L000400	3,227896225	0,2429	2,320669	4,135123
128	TN8.4	3,257656693	0,24311	2,349642	4,165672
129	L000216	3,259389629	0,29397	2,162331	4,356448
130	Sephi	3,25992942	0,24311	2,351914	4,167944
131	L000134	3,263687081	0,24287	2,356544	4,17083
132	L000263	3,26650196	0,24305	2,358694	4,17431
133	TN8.23	3,270141551	0,24317	2,361884	4,178399
134	L000337	3,2743132	0,21314	2,477254	4,071373
135	L000356	3,279644673	0,24353	2,370021	4,189268
136	L000314	3,288906873	0,21313	2,491878	4,085935
137	L000303	3,294014525	0,24364	2,383986	4,204043
138	L000321	3,305919287	0,24364	2,395891	4,215948
139	L000322	3,30604379	0,24304	2,398283	4,213805
140	L000451	3,314152523	0,24285	2,407089	4,221216

	Line	MSS Lsmean	SE	lower.CL	upper.CL
141	L000343	3,31609624	0,2429	2,40884	4,223353
142	L000340	3,318140492	0,2429	2,410884	4,225397
143	L000450	3,32830573	0,24286	2,421221	4,23539
144	L000549	3,355325932	0,24286	2,448241	4,262411
145	L000178	3,358000263	0,24361	2,448105	4,267895
146	L000290	3,360687949	0,24294	2,453293	4,268083
147	L000368	3,372762167	0,24361	2,462867	4,282657
148	L000049	3,379825659	0,24361	2,46993	4,289721
149	L000371	3,384806039	0,21313	2,587782	4,18183
150	L000309	3,389689352	0,24296	2,482241	4,297138
151	L000448	3,392337301	0,24285	2,485274	4,299401
152	L000520	3,410487779	0,24283	2,50351	4,317465
153	TN9.21	3,412880211	0,24328	2,504206	4,321555
154	L000198	3,413555818	0,24361	2,503661	4,323451
155	L000173	3,41863701	0,24302	2,510956	4,326318
156	L000438	3,419509666	0,24285	2,512446	4,326573
157	L000215	3,42312057	0,21296	2,626727	4,219514
158	L000306	3,426693131	0,24291	2,519411	4,333975
159	L000649	3,433224724	0,24298	2,5257	4,340749
160	L000226	3,441140183	0,24287	2,533997	4,348283
161	TN8.3	3,445115824	0,24274	2,538483	4,351749

	Line	MSS Lsmean	SE	lower.CL	upper.CL
162	TN7.4	3,47595941	0,24325	2,567407	4,384512
163	L000555	3,478071724	0,24279	2,571246	4,384897
164	L000225	3,485793206	0,24282	2,578839	4,392747
165	L000286	3,487420593	0,24303	2,579681	4,39516
166	L000601	3,487581181	0,24353	2,577958	4,397205
167	TN1.18	3,493178617	0,24328	2,584504	4,401853
168	L000147	3,503853858	0,24287	2,596711	4,410997
169	TN7.19	3,508426943	0,24325	2,599874	4,41698
170	L000162	3,541096008	0,24273	2,634509	4,447683
171	L000449	3,541392709	0,24287	2,63425	4,448536
172	L000530	3,555555556	0,1337	3,046265	4,064846
173	TN9.22	3,555917439	0,24284	2,64892	4,462915
174	L000545	3,578328945	0,21279	2,782562	4,374096
175	TN8.22	3,579579646	0,24325	2,671027	4,488132
176	TN8.25	3,579741673	0,24317	2,671484	4,487999
177	L000313	3,584443002	0,24277	2,677711	4,491175
178	TN8.21	3,596810781	0,24325	2,688258	4,505364
179	L000148	3,596934991	0,24294	2,689551	4,504319
180	L000365	3,599385846	0,2429	2,692159	4,506613
181	L000376	3,600502654	0,24291	2,693221	4,507785

	Line	MSS Lsmean	SE	lower.CL	upper.CL
182	L000574	3,603669597	0,24283	2,696692	4,510647
183	L000052	3,608169134	0,21313	2,811145	4,405193
184	L000375	3,618597402	0,24295	2,711167	4,526027
185	L000651	3,628193717	0,21316	2,83105	4,425337
186	L000522	3,629231656	0,24286	2,722147	4,536316
187	L000537	3,629723434	0,24291	2,722441	4,537006
188	L000386	3,630747039	0,24285	2,723684	4,53781
189	D3.3.3	3,634471645	0,24286	2,727398	4,541545
190	L000202	3,635726799	0,24294	2,728332	4,543121
191	L000219	3,648464403	0,24286	2,74136	4,555569
192	L000232	3,648593942	0,24305	2,740786	4,556402
193	TN9.17	3,664784408	0,24317	2,756527	4,573042
194	L000239	3,667722485	0,24361	2,757827	4,577618
195	L000550	3,67056556	0,2131	2,873651	4,46748
196	L000552	3,678533346	0,24287	2,77139	4,585676
197	L000458	3,685006816	0,24297	2,777494	4,59252
198	L000332	3,687049783	0,24295	2,77962	4,59448
199	L000543	3,692709319	0,24279	2,785886	4,599533
200	L000679	3,70934336	0,29534	2,607138	4,811549
201	L000167	3,716140183	0,24287	2,808997	4,623283
202	D4.2.1	3,718199619	0,24307	2,810327	4,626073

	Line	MSS Lsmean	SE	lower.CL	upper.CL
203	L000383	3,724345398	0,2429	2,817089	4,631602
204	L000165	3,724615688	0,24294	2,817221	4,63201
205	L000228	3,740227919	0,24302	2,832547	4,647909
206	D6.2.1	3,741116286	0,24307	2,833243	4,648989
207	L000126	3,743088599	0,24364	2,83306	4,653117
208	L000246	3,751923013	0,29375	2,65568	4,848166
209	L000280	3,762327617	0,24277	2,855595	4,66906
210	L000270	3,76403287	0,24303	2,856293	4,671772
211	L000475	3,772524816	0,24283	2,865547	4,679502
212	L000529	3,774997669	0,24294	2,867613	4,682382
213	D1.2.3	3,780784902	0,24307	2,872912	4,688658
214	L000350	3,790342999	0,24285	2,88328	4,697406
215	L000639	3,791866896	0,24353	2,882243	4,70149
216	L000174	3,79377692	0,24362	2,883844	4,70371
217	L000557	3,793917961	0,24287	2,886775	4,701061
218	D2.2.2	3,794290889	0,24307	2,886418	4,702164
219	L000217	3,795853108	0,24298	2,888329	4,703377
220	L000470	3,800973148	0,2429	2,893746	4,7082
221	L000344	3,803843823	0,24294	2,896459	4,711228
222	L000163	3,811832476	0,24362	2,901899	4,721766

	Line	MSS Lsmean	SE	lower.CL	upper.CL
223	D5.3.1	3,817523174	0,24286	2,91045	4,724596
224	L000154	3,818482392	0,19275	3,096545	4,54042
225	L000387	3,821285682	0,24286	2,914202	4,728369
226	L000680	3,821676179	0,24267	2,915311	4,728041
227	L000362	3,828255784	0,24353	2,918632	4,737879
228	L000355	3,829644673	0,24353	2,920021	4,739268
229	L000238	3,838763426	0,24296	2,931315	4,746212
230	L000455	3,840098813	0,24286	2,933016	4,747182
231	D7.1.3	3,854112169	0,24298	2,946588	4,761637
232	A20	3,874600537	0,24279	2,967768	4,781433
233	L000659	3,875298391	0,24298	2,967774	4,782823
234	Caliph	3,8892994	0,24317	2,981042	4,797557
235	L000650	3,892172812	0,24267	2,985808	4,798538
236	L000360	3,903294618	0,21314	3,106238	4,700351
237	L000307	3,919014525	0,24364	3,008986	4,829043
238	L000276	3,954019959	0,24286	3,046915	4,861125
239	L000354	3,957109763	0,24364	3,047082	4,867138
240	L000267	4,050638695	0,24294	3,143254	4,958023
241	L000379	4,085681191	0,24364	3,175653	4,995709
242	L000283	4,090338661	0,24304	3,182578	4,9981
